# Molecular Characterization and Evolutionary Analyses of *Carnivore Protoparvovirus 1* *NS1* Gene

**DOI:** 10.3390/v11040308

**Published:** 2019-03-29

**Authors:** Francesco Mira, Marta Canuti, Giuseppa Purpari, Vincenza Cannella, Santina Di Bella, Leonardo Occhiogrosso, Giorgia Schirò, Gabriele Chiaramonte, Santino Barreca, Patrizia Pisano, Antonio Lastra, Nicola Decaro, Annalisa Guercio

**Affiliations:** 1 Istituto Zooprofilattico Sperimentale della Sicilia “A.Mirri”, Via Gino Marinuzzi n. 3, 90129 Palermo, Italy; dottoremira@gmail.com (F.M.); giuseppa.purpari@izssicilia.it (G.P.); vincenza.cannella@izssicilia.it (V.C.); giorgia.schiro@hotmail.it (G.S.); gabrielechiaramonte90@gmail.com (G.C.); santinobarreca@gmail.com (S.B.); pisano.patrizia@libero.it (P.P.); lastra.antonio77@gmail.com (A.L.); annalisa.guercio@izssicilia.it (A.G.); 2 Department of Biology, Memorial University of Newfoundland, 232 Elizabeth Ave., St. John’s, NL A1B 3X9, Canada; marta.canuti@gmail.com; 3 Department of Veterinary Medicine, University of Bari, Strada provinciale per Casamassima Km 3, 70010 Valenzano, Italy; leonardo.occhiogrosso@uniba.it (L.O.); nicola.decaro@uniba.it (N.D.)

**Keywords:** *Carnivore protoparvovirus 1*, canine parvovirus, feline panleukopenia virus, NS1, NS2, sequence analysis, evolution

## Abstract

*Carnivore protoparvovirus 1* is the etiological agent of a severe disease of terrestrial carnivores. This unique specie encompasses canine parvovirus type 2 (CPV-2) and feline panleukopenia virus (FPLV). Studies widely analyzed the main capsid protein (VP2), but limited information is available on the nonstructural genes (*NS1*/*NS2*). This paper analyzed the *NS1* gene sequence of FPLV and CPV strains collected in Italy in 2009–2017, along with worldwide related sequences. Differently from VP2, only one NS1 amino-acid residue (248) clearly and constantly distinguished FPLV from CPV-2, while five possible convergent amino-acid changes were observed that may affect the functional domains of the NS1. Some synonymous mutation in NS1 were non-synonymous in NS2 and vice versa. No evidence for recombination between the two lineages was found, and the predominance of negative selection pressure on NS1 proteins was observed, with low and no overlap between the two lineages in negatively and positively selected codons, respectively. More sites were under selection in the CPV-2 lineage. NS1 phylogenetic analysis showed divergent evolution between FPLV and CPV, and strains were clustered mostly by country and year of detection. We highlight the importance of obtaining the *NS1*/*NS2* coding sequence in molecular epidemiology investigations.

## 1. Introduction

*Carnivore protoparvovirus 1* is a member of the *Protoparvovirus* genus (family *Parvoviridae*, subfamily *Parvovirinae*). As defined by the International Committee on Taxonomy of Viruses (ICTV) [1], both canine parvovirus type 2 (CPV-2) and feline panleukopenia virus (FPLV) are included in this unique specie, together with mink enteritis virus (MEV) and raccoon parvovirus (RaPV) [2,3]. In susceptible dogs and cats, *Carnivore protoparvovirus 1* commonly causes an acute and often lethal disease, inducing vomiting, enteritis, diarrhea, and acute lymphopenia [4].

FPLV is known since the beginning of the 20th century [5] and, during decades, it maintained a certain genetic stability [6]. Contrarily, CPV-2 emerged as a dog pathogen only in the late 1970s, most likely as a host variant of the feline virus or a related strain [7], and it displayed higher rates of nucleotide changes [8,9,10,11]. Indeed, soon after its emergence, the original CPV-2 type was replaced by two antigenic variants, CPV-2a and 2b [12,13], and in 2000 a third variant was detected, termed CPV-2c [14]. While dogs are susceptible only to the original CPV-2 and all its variants, cats are susceptible to both FPLV and CPV-2 variants [15,16,17], except the original CPV-2 type, despite the fact that FPLV remains the prevalent cause of parvovirus infection in domestic felines [18].

*Carnivore protoparvovirus 1* includes small, non-enveloped, linear single-stranded DNA viruses. Their genome consists of an approximately 5200-nucleotide (nt) DNA molecule containing two large open reading frames (ORFs), encoding for two nonstructural (NS1 and NS2) and for two structural (VP1 and VP2) proteins, generated through alternative splicing of the same messenger RNAs (mRNAs) [4,19].

Among encoded structural proteins, VP2 is the major capsid protein, represents the main determinant of host range, and is subject to antibody-mediated selection [20,21]. On the other hand, the nonstructural proteins NS1 and NS2 are essential for viral replication, DNA packaging, cytotoxicity, and pathogenicity [22,23,24]. Due to the involvement of the VP2 capsid protein in host switch and due to its fast evolutionary rate, most studies on CPV and FPLV focused their attention on the *VP2* gene and on the structural analysis of the encoded protein. Contributions on the genetic analysis of the nonstructural genes [18,24] and on the structural analysis of the encoded proteins are limited, highlighting the limits of available sequence data [8,25,26,27,28]. The nonstructural protein 2 (NS2) of FPLV and CPV is produced by the conjunction of left-hand 260-nt and right-hand 238-nt genetic fragments of the NS1 open reading frame, but no studies were conducted on the NS2 amino-acid divergences within this viral species.

Only a few studies described the dynamics driving genetic changes of the CPV and FPLV *NS1* gene and the potential recombination events involving this gene [29,30], although recombination was hypothesized as a potential alternative source of genetic variations [18]. Moreover, the *NS1* gene molecular features, as a useful tool in outbreak tracing, were only recently reconsidered [31,32]. Despite the most recent sequence analyses on CPV also including this genomic ORF, there are still limited studies on the FPLV NS1 sequence [30,33,34,35].

The aim of this paper was to characterize NS1 sequences of FPLV and CPV strains, collected from cats and dogs in Italy, and to compare them to NS1 sequences available in public domain sequence databases. Sequence analyses, genetic diversity estimation, evaluation of potential recombination events, and phylogeny studies were performed to better elucidate the molecular features of NS1 and its role in the evolution of CPV-2 and FPLV. Moreover, the NS2 sequences were also characterized and the deduced amino-acid divergences were analyzed.

## 2. Materials and Methods

### 2.1. Sample Collection

The sequences analyzed in this study were obtained from samples collected from 18 cats and 29 dogs from 2009 to 2017. Samples or carcasses of animals with suspicion of parvovirus infection were submitted to the Istituto Zooprofilattico Sperimentale della Sicilia “A. Mirri” (Palermo, Italy) for necropsy for diagnostic purposes. Rectal swabs or organs (intestine, spleen, heart, brain) from domestic dogs and cats with different geographical origins and living conditions (client-owned or shelter animals) were collected and submitted for virological analyses. Details are summarized in Table 1.

### 2.2. DNA Extraction and Parvovirus PCR

Viral DNA was extracted from 200 μL of swab/organ homogenate, obtained as previously described [37], using a DNeasy Blood and Tissue Kit (Qiagen S.p.A., Milan, Italy) according to the manufacturer’s instructions. Presence of FPLV and CPV DNA was evaluated using a primer pair amplifying a 700-bp fragment of the *VP2* gene [38] following a previously described PCR protocol [32].

### 2.3. Viral Isolation

Samples that tested positive (in bold in Table 1) were processed as previously described [37] and supernatants were inoculated in 80% confluent cell (A-72, CrFK) monolayers, cultured in minimum essential medium (MEM) with 10% bovine fetal serum (EuroClone S.p.A., Pero, Italy), antibiotic and antifungal solution (100 U/mL penicillin G sodium salt, 0.1 mg/mL streptomycin sulfate, 0.25 ug/mL amphotericin B; PAA Laboratories GmbH, Austria), 1% sodium pyruvate (A-72 cells), and 0.1% lactalbumin (CrFK cells). Inoculated cells were daily monitored for a maximum of five days and viral growth was evaluated by detection of cytopathic effect (CPE) and PCR. A total of five passages were carried out before considering virus isolation as unsuccessful.

### 2.4. Sequence Analysis

CPV/FPLV DNA from positive samples and from cell cultures with CPE were submitted to sequencing. Analyses were conducted amplifying a long genomic sequence, encompassing both ORFs, NS and VP, using primers pairs described by Pérez et al. [28] and the commercial kit GoTaq^®^ G2 DNA Polymerase (Promega Italia s.r.l., Milan, Italy). Reaction mixes were prepared as previously described [36], with minor modifications (thermal conditions: 2 min for the elongation steps). Positive amplicons were purified with Illustra^TM^ GFX^TM^ PCR DNA and Gel Band Purification Kit (GE Healthcare Life Sciences, Amersham, Buckinghamshire, UK) and submitted to BMR Genomics srl (Padua, Italy) for direct Sanger sequencing. Overlapping sequences were assembled using BioEdit ver. 7.2.5 software [39] and a nearly complete genomic sequence for each sample was obtained.

Ten positive cell culture supernatants were submitted to the Istituto Zooprofilattico Sperimentale della Lombardia e dell’Emilia Romagna “Bruno Ubertini” (Parma, Italy) for sequencing service using next-generation sequencing (NGS) methodologies. DNA was extracted using the One for All Vet Kit (Qiagen, Milan, Italy) and amplified using primers F194/NS-Rext and 2161F/R4848 described by Pérez et al. [28]. Sequencing libraries were prepared using the Nextera XT kit (Illumina Inc. San Diego, CA, USA) and sequenced using the Illumina MiSeq (Illumina Inc. San Diego, CA, USA) system. Read files generated by the sequencer were assembled and analyzed using the software SeqMan NGen 12.0.0 (DNASTAR, Madison, WI, USA).

The complete nucleotide *NS1* coding sequences (2007 nt) alignments were obtained using the ClustalW program included in the BioEdit software. Sequences were submitted to nBLAST [40] to search related sequences in public domain databases. In December 2017, 26 FPLV and 141 CPV complete *NS1* sequences were obtained from NCBI database, including two FPLV and 18 CPV sequences previously submitted to the same database from the Istituto Zooprofilattico Sperimentale della Sicilia “A. Mirri”. Sequences originated from samples collected in years 1964–2016 (FPLV) and 1978–2017 (CPV), from domestic and wild animals in America (nine FPLVs and 34 CPVs from North America; 41 CPVs from South America), Europe (two FPLVs; three CPVs), Asia (14 FPLVs; 57 CPVs), and Oceania (one FPLV; four CPVs) (see Dataset S1, Supplementary Materials).

*NS1* and *VP2* gene sequences were aligned with reference sequences obtained from the NCBI database, translated into amino-acid (aa) sequences (668 and 584 aa, respectively), and analyzed using the BioEdit software. The complete nucleotide *NS2* coding sequences (498 nt) were also obtained from the whole dataset of sequences using the ClustalW program and analyzed using the BioEdit software. Viral typing was based on the analysis of VP2 amino-acid (aa) residues discriminating the viral type (FPLV/CPV) and the CPV variants [41]. Sequence data were submitted to the DDBJ/EMBL/GenBank databases under accession numbers reported in Table 1.

### 2.5. Recombination and Selection Pressure Analyses

The NS1 alignment was tested for the presence of potentially recombinant sequences with all the different methods included in the RDP 4 software package [42], as described in Canuti et al. [43]. Detected recombination events were confirmed by constructing maximum-likelihood phylogenetic trees with MEGA7 software [44], inferred with the maximum-likelihood method based on the Hasegawa–Kishino–Yano and Kimura 2-parameter models [45,46], the best-fitting models after the model test analysis. A discrete Gamma distribution was used to model evolutionary rate differences among sites.

To estimate the presence of selection pressure, the overall average synonymous (d*S*) and non-synonymous (d*N*) substitutions for each alignment (whole dataset, CPV subset, and FPLV subset) were calculated with the *Z*-test of selection implemented in MEGA 7. The Nei–Gojobori method [47] was used to test hypotheses of deviation from strict neutrality (null hypothesis, d*N* = d*S*), test of neutrality (d*N* =/= d*S*), purifying selection pressure (d*N* < d*S*), and positive selection pressure (d*N* > d*S*). Variance was estimated with the bootstrap method and 1000 replicates. 

Individual sites under positive and purifying selection were identified with FUBAR (Fast Unconstrained Bayesian Approximation for inferring selection) [48], while those under episodic diversifying selection were detected with MEME (Mixed-Effects Model of Evolution) [49]. Sites under selection were considered acceptable only when statistically significant (*p* < 0.1 for MEME and posterior probability >0.9 for FUBAR). Both methods are available on the Datamonkey Adaptive Evolution Server (https://www.datamonkey.org). FUBAR and MEME were performed on the FPLV and CPV branches separately, after excluding potentially recombinant sequences.

### 2.6. Phylogenetic Analysis

To elucidate the genetic relationships between the obtained CPV and FPLV strains and the reference sequences, a phylogenetic tree was constructed. Due to the high number of sequences in dataset 2, a subset of 86 sequences was generated by excluding highly identical or identical sequences derived from the same geographic area and the same year. The model selection was performed using the best-fit model of nt substitution with MEGA7 software [44]. A phylogenetic tree was constructed with the MEGA7 software using the maximum-likelihood (ML) method according to the Hasegawa–Kishino–Yano model [45] with discrete Gamma distribution (five rate categories) and bootstrap analyses with 1000 replicates. Viral type or CPV variants, based on the analysis of the VP2 aa residues as described above, were depicted in the phylogenetic tree for each NS1 sequence. Depicted clades and subclades in the phylogenetic tree were numbered with Roman numerals and are not meant as a classification of the type/variants, but rather to allow easier referencing in the text.

For comparison, a phylogenetic tree based on the *VP2* gene sequences of the same strains included in the NS1 tree was constructed with the MEGA7 software using the ML method according to the Tamura three-parameter model [45] with discrete Gamma distribution (five rate categories) and bootstrap analyses with 1000 replicates. 

## 3. Results

### 3.1. Detection and Characterization of FPLV and CPV

All samples analyzed tested positive for *Carnivore protoparvovirus 1*. Positive samples were obtained from tissues commonly known as viral targets, such as intestine, spleen, and lymph nodes, as well as less tested tissues such as brain and cerebellum [50]. Based on the analysis of the VP2 amino-acid residues, 17 strains from the 18 cats were typed as FPLV and one was typed as CPV-2c. Among the samples collected from the 29 dogs, 13, one, and 15 strains were typed as CPV2a, CPV-2b, and CPV-2c variants, respectively. The CPV-2c strain collected from the cat (sample identifier PA15423/16) showed high identity with the other CPV-2c strains from this study collected from dogs (NS1: 100–99.75%; VP2: 99.94–99.71%) and the highest identity rates with the strain 41113c1/16, collected in the same year. Viral types/variants are listed in Table 1.

Interestingly, two unreported amino-acid changes were observed within the FPLV *VP2* gene sequences; the aa change A359G was also observed in two FPLV VP2 sequences (accession number KY083101–KY083104) from Singapore in 2015, and the aa change D311N, which was unique to the strains analyzed in this study, was never reported in other FPLV sequences. VP2 non-synonymous changes of analyzed FPLV and CPV strains are listed in Table S2 (Supplementary Materials).

### 3.2. Sequence Analysis of NS1 Gene

The *NS1* gene sequences of 2007 base pairs in length were obtained from each sample. Among FPLV *NS1* sequences, 17 nucleotide substitutions were observed, resulting in 12 synonymous and five non-synonymous (81V/I, 115I/V, 247H/Q, 595H/Q, 664Q/R) changes (Table 2).

Among the CPV *NS1* sequences, 63 nucleotide substitutions were observed, resulting in 52 synonymous and 11 non-synonymous (60I/V, 239N/T, 350D/N, 397L/F, 544Y/F, 545E/V, 572K/E, 584T/A, 590P/S, 597L/P, 630L/P) changes (Table 3).

Comparison of the analyzed sequences with those from the NCBI database evidenced only one aa change clearly distinguishing FPLV from CPV strains; at residue 248, FPLV showed T while CPV showed I, due to a nucleotide change in the second base of the codon (c743t).

Other differences among CPV and FPLV strains were found at aa residues 247, 545, and 595 (Table 2 and Table 3; Dataset S1, Supplementary Materials). All CPV sequences showed a Q at residues 247 and 595 and an E at residue 545. The only exceptions were some strains of Asian origin, which showed V at residue 545. In contrast, FPLV sequences showed an H at residues 247 and 595, and a Q at residue 545. Unlike most of the FPLV sequences, nine analyzed FPLV sequences (Table 2; Dataset S1, Supplementary Materials) evidenced residues identical to CPV-2 at these sites (Q at residues 247 and 595, and E at residue 545). Change Q545E was evidenced in all FPLV strains collected in Italy.

In some FPLV sequences obtained from this study, two additional changes (V115I and R664Q) were evident (Table 2) that were previously observed only in three CPV sequences from China or Vietnam (Dataset S1, Supplementary Materials).

Only old FPLV strains from domestic/wild felids in the United States of America (USA) (M38246, EU659111, EU65913-15), Japan (AB000048-49, AB000057, AB000060, AB000062), and more recently from wild carnivores in Canada (MF069445-47) showed the specific changes N23D, V165I, and I443V (Dataset S1, Supplementary Materials).

Differences among CPVs were observed in strains collected in Italy in 2016–2017 (Table 3). Change D350N was also observed in older sequences (1983–2008), almost all collected in the USA. Changes Y544F and L597P were observed both in older sequences from the USA (1983–2010) and New Zealand (1994), as well as in more recent sequences from South America, Canada, and China.

Among sequences from CPV-2c strains, specific residues were observed in different geographic areas, such as Australia (11K, 25P, 72K, 73K, 74K), Uruguay and Brazil (351K), China and Vietnam, and in an Italian imported dog (MF510157) (60V, 630P). 

### 3.3. Sequence Analysis of NS2 Gene

Nucleotide substitutions resulting in some non-synonymous changes in *NS1* also lie in the NS2-encoding sequence (V81I and H595Q, and I60V, T584A, P590S, L597P, and L630P of the FPLV and CPV NS1 sequences, respectively), while changes at codons 597 and 630 of *NS1* CPV-2 sequences did not result in any changes in the NS2 protein. Other changes generated the additional aa changes V81I and S105R, and 60I/V, D93G, and S99F in the NS2 of FPLV and CPV sequences, respectively (Dataset S3, Supplementary Materials).

Other amino-acid divergences in the CPV NS2-enconding sequences were observed among the analyzed strains: 94T/A, 109S/F, 110D/N, 151N/D, and 160E/Q. These changes were synonymous in the corresponding NS1 amino-acid residues. All these changes in the NS2 sequences are summarized in Dataset S3 (Supplementary Materials).

Divergences between FPLV and CPV were observed at aa residues 152 and 163. At residue 152, an M was observed in FPLV strains from the USA, Canada, Japan, and Australia, and a V was observed in the most recent FPLV strains from Europe (Italy and Belgium) and China. On the other hand, amino acid V was observed in almost all CPV strains, with the exception of old CPV-2 and CPV-2a strains from the USA, which showed M at the same residue. At residue 163, the FPLV strains, including the strains collected in Italy, showed the amino acid L, with the exception of some strains which showed F at the same residue. These changes in the *NS2* sequence resulted in silent mutations in the corresponding aa residues of the NS1 sequence of the same strains (aa 642 and 653).

### 3.4. Recombination and Selection Pressure Analyses

The analysis performed with RDP identified only one potential recombination event, involving three CPV sequences (recombinant: CPV-2a, KT382542; major parent: CPV-2b, KP749859; minor parent: CPV-2c, KP749873) (Recombination and Selection Pressures S4, Supplementary Materials), with one breakpoint (nt 1515), and this was confirmed by phylogenetic reconstructions (Recombination S5, Supplementary Materials). Therefore, as positively selected sites may be overestimated when recombination is present [51], all selection pressure analyses were performed on the entire alignment after excluding the potentially recombinant sequence.

The *Z*-test allowed us to reject the null hypothesis of strict neutrality and detected an overall presence of purifying selection in all cases. FUBAR identified 124 and 42 sites to be under negative selection pressure in the CPV and FPLV clades, respectively (Table 4), and only nine and two of these sites were located within the helicase motives [23]. Only 13 sites were found to be under negative selection pressure in both clades (in bold in Table 4). Within the CPV clade, FUBAR identified five sites (19, 278, 545, 572, and 583) to be under pervasive positive selection and MEME identified two additional sites (597 and 647) under episodic positive selection. Interestingly, in correspondence of the positively selected site 545, a case of convergent mutation between the CPV and FPLV clades (mutation C to G in some FPLV strains) was identified. Finally, only one site (443) resulted as being subjected to pervasive positive selection in the FPLV clade. This site is located within the Walker motif B of the NS1 helicase domain (440 to 445, LIW(I/V)EE) and, whereas all analyzed CPV strains had the amino-acid I in this position, 13 out of 43 of the analyzed FPLV strains possessed a V. However, 443V was identified mainly in viruses from older cats (1967 to 1995 and only one in 2006) and in raccoons from a segregated environment [34].

### 3.5. Phylogeny

**Figure 1:**
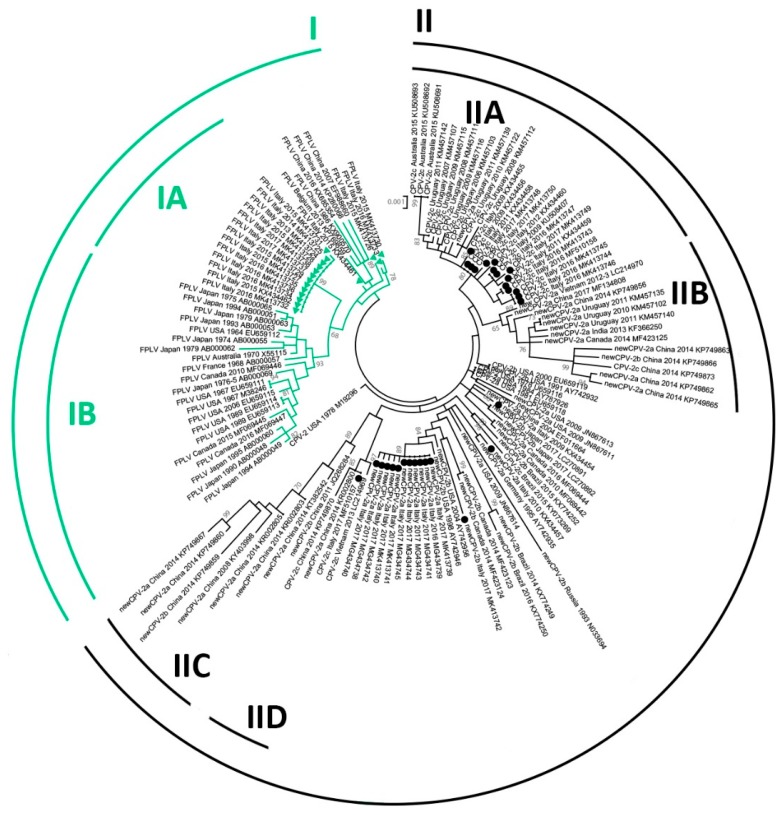
Maximum-likelihood tree based on 133 complete *NS1* gene sequences of feline panleukopenia virus (FPLV, in green) and canine parvovirus type 2 (CPV-2, in black) strains (bootstrap 1000 replicates; bootstrap values greater than 65 are shown). Green triangles and black dots indicate, respectively, FPLV and CPV strains analyzed in this study. Each sequence is indicated with virus type (FPLV/CPV) or variant (CPV-2, CPV-2a, CPV-2b, CPV-2c), country and year of collection, and accession number. The term “new” was used to distinguish the CPV-2a/2b strains with S297A main capsid protein (VP2) amino-acid changes from the early CPV-2a/2b variants. The same tree with more information about used strains (strain/isolate name) is available in Figure S6 (Supplementary Materials).

Figure 1 shows the phylogenetic tree inferred from NS1 sequences. Unfortunately, likely because of high sequence identity, obtained bootstrap values were sometimes poor and only bootstrap-supported sub-clades are indicated in the Figure. For clarity, only viral type, origin and year of detection, and accession number of strains are reported, while the same tree with the full strain information is available in Figure S6 (Supplementary Materials). Although with a low support (bootstrap = 49), the sequences analyzed in this study clustered in separate clades according to the type of the virus (CPV/FPLV), but not the CPV variant. Indeed, CPV-2 strains (indicated in black in the figure) tended to segregate according to the country and the year of collection rather than according to the strain variant.

Most of the CPV-2c variants, with the exception of two strains (KP749873, KR002800), clustered in two distant subclades: the IIA subclade, in which strains collected in Italy, Uruguay, and Australia were included, and the IID subclade, which included strains with Asian origin. The CPV-2a/2b sequences collected from China were located together (subclade IIC) and were close to the CPV-2c strains of Asian origin (subclade IID), although the bootstrap support for this clade was low (bootstrap = 47). However, a common geographic origin was not maintained in the IIB subclade, which included CPV-2a/2b/2c viruses collected in Asia, Uruguay, and Canada.

Within clade I, including the FPLV strains, most of the recent Italian FPLV strains and the oldest FPLV strains clustered in statistically supported subclades (IA and IB, respectively), separately from those with convergent aa changes with CPV, which were located close to the CPV clade (between CPV and subclades IA/IB). 

Figure S7 (Supplementary Materials) shows the phylogenetic tree inferred from the VP2 sequences of the same strains. Similar to the NS1 tree, the separation of the two main lineages was highly supported, whereby the FPLV sequences with convergent NS1 mutations were located closer to the CPV-2 clade, and geographic segregation of strains was partial. Finally, although without support, the CPV-2c sequences were separated from other strains. 

## 4. Discussion

Almost all molecular studies on *Carnivore protoparvovirus 1* coding genes focused on the analysis of the *VP2* gene. The studies on the VP2 evolutionary dynamics helped clarify the spread of *Carnivore protoparvovirus 1* species, particularly after the first appearance of CPV-2. The VP2 protein determines the host range and antigenicity and, indeed, clear descriptions of the coding aa residues, which determine these features, were used to elucidate the host species jump from felids to canids [52,53,54,55,56]. Therefore, the separate evolution of FPLV and CPV was explained probably due to the different degree of the evolutive driving forces and to the different mutation rates among the viral types [8,57]. Moreover, studies based on the VP2 sequences evidenced the genetic stability of FPLV [11] and the continuous appearance of genetic mutants in CPV-2 [6,36,58]. In the current literature, there are only a few studies on the FPLV and CPV nonstructural genes [18], also due to the limited availability of updated *NS* genetic sequences for comparison [28], despite their essential role for viral replication, cytotoxicity, and pathogenicity [23,59,60]. To expand the current knowledge on the evolution of the *Carnivore protoparvovirus 1* members, this study provided a molecular characterization and evolutionary analysis of NS1 and NS2, comparing sequences of FPLV and CPV obtained from cats and dogs.

Early studies based on the comparison among only four FPLV and CPV NS1 sequences [61] evidenced a lesser degree of conservation of the NS1 aa sequence of CPV compared to FPLV, and 13 aa changes among the sequences of these viruses were described. Subsequent studies, based on few available sequences, detected only five or three aa differences in the NS1 protein between FPLV and CPV [25,26]. In the present study, all the distinctive aa residues between FPLV and CPV viral types in the *VP2* gene [41] were maintained; however, in the NS1-encoding sequence, only one amino-acid residue (248) clearly and constantly distinguished FPLV from CPV. Indeed, sequence analysis demonstrated that amino acids at specific residues (247, 545, and 595), previously potentially designated as discriminating the viral types, were present in nine FPLV strains collected in Italy, China, and Belgium, as well as in all the available CPV sequences. Previously, potential recombination events were hypothesized for some FPLV strains collected in China and Belgium [29,30,33,35] on the basis of the evidence of potential breakpoints located between the *NS1* and the *VP2* gene sequences, and of specific CPV-2 aa residues found in the NS1 sequence of FPLV strains. In this study, no evidence for recombination between FPLV and CPV was found within the NS1 sequence, although a larger sequence dataset in comparison to previous studies was evaluated. Moreover, specific amino acids supposed to be characteristic of CPV (247Q, 545E, and 595Q) were also observed among FPLV strains. Similarly, other substitutions (V115I and R664Q) emerged more recently both in FPLV and in CPV strains but in separate environments. These observations led us to conclude that these changes are more probably due to convergent substitutions that emerged independently in the two lineages (FPLV/CPV) rather than as a result of recombination events. According to these data, recombination does not seem to play an important role in shaping the evolution of the *NS1* gene of CPV and FPLV, as our dataset contained only one potentially recombinant sequence and no evidence for recombination between FPLV and CPV was found. However, due to the high sequence identity between these two viruses, it is possible that some recombination events went undetected and the importance of recombination may have been underestimated.

The circulation of CPV-2 variants in cats [15,16,17,62] arose questions about the epidemiological role of this species in parvovirus ecology [63] and suggested that cats may act as a potential source of new parvovirus variants [57]. Indeed, superinfection and co-infection with different species of parvovirus in the feline host led to a high genetic variability and the potential emergence of new viruses [57]. Unfortunately, no NS1 sequence is available for comparison with strains associated to superinfections or co-infections.

Moreover, our data do not confirm the detection of aa change L582S in the FPLV strains recovered from the central nervous system compared to strains collected in other tissues [35]. All samples from cerebral tissue of dogs and cats tested positive for *Carnivore protoparvovirus 1*, but no specific amino acid change was observed in any sample. This change occurred rather in CPV strains in the spleen tissues of coyotes in Canada in 2014 [34] and not in any other sequence of the analyzed dataset. 

Several differences were found when studying selection pressure forces acting on the NS1 of CPV and FPLV. Firstly, considerably more amino acids were identified to be both positively and negatively selected in the CPV lineage compared to the FPLV lineage (7.0 and 3.4 times more, respectively). However, as only a limited number of FPLV sequences are currently available, it is possible that the wide FPLV diversity was not well represented in our dataset and, therefore, the number of positively and negatively selected site was underestimated. However, our results are consistent with those of previous studies that identified FPLV to be more stable and less subjected to positive selection [11,25].

Nevertheless, when we compared the sites that were subjected to selection pressure forces, surprisingly, there was only a minimal overlap between the two viruses. Only 13 negatively selected codons were identified in both viruses, while the vast majority of negatively selected codons were identified in either FPLV or CPV lineage (about 69% and 89.5%, respectively) and there was no overlap for positively selected codons. Our analyses evidenced a predominance of negative selection pressure on nonstructural proteins, slightly more marked among CPV strains. Interestingly, whereas two different amino acids could be observed among FPLV strains at residue 443 (V: 30%, I: 70%), within one of the functional domains (Walker B) of the viral helicase, only amino acid I was identified in all CPV strains. Although these two amino acids have very similar properties [64], keeping variation at this site might be important for the overall viral fitness. However, these findings need to be confirmed by specific mutagenesis studies.

Our results suggest that NS1 might be subjected to different evolutionary dynamics within the two lineages. Furthermore, the reduced viral diversity within the FPLV lineage (mean within-group distance: 0.6% for FPLV and 0.8% for CPV) could also reflect a different evolutionary behavior of these viruses. FPLV has been circulating within the feline population for a long time, whereas the CPV pandemic originated only recently and this virus was shown to evolve fast within the canine population [13]. This could partially explain our results. Furthermore, different evolutionary dynamics could be explained by the different infection dynamics and replication efficiency of the two viruses in the feline and the canine hosts. In fact, a more sustained transmission and, therefore, a shorter generation time favor a faster evolution [65]. These hypotheses highlight the need of acquiring the NS1 sequence of more strains in order to obtain a larger dataset, which is necessary to confirm our findings.

**Figure 2:**
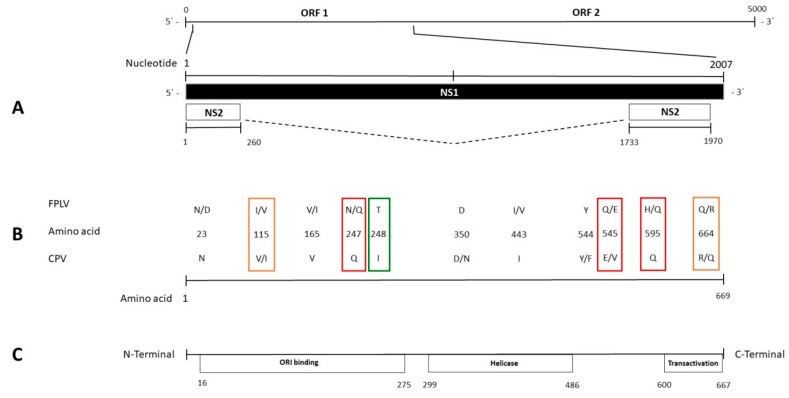
Schematic representation of the FPLV and CPV genome. The scheme, in the upper lines (**A**), represents the complete nucleotide (nt) length of the genome from the 5’ to the 3’ UTR and of the *NS1* and *NS2* genes. The relative positions of the described amino-acid changes in the *NS1* gene sequence are indicated in the middle lines (**B**). The lower line (**C**) represents the location of the potential functional domains within the *NS1* gene sequence, extended between the depicted amino-acid residues. Colored squares highlight the amino-acid changes distinguishing FPLV from CPV strains (green) and the convergent amino-acid changes between FPLV and CPV identified in the whole dataset (red) or only among the Italian FPLV strains (orange).

There are also limited studies on the NS1 protein, based only on evaluations of its functions [66,67,68] and on the potential location of its functional domains [10,22,23]. Changes in specific residues of NS1 CPV sequence [27] or affecting functional domains in others *Carnivore protoparvovirus 1* such as mink enteritis parvovirus [69] were hypothesized as potentially affecting the functions of this protein. According to a previous study [22], several described changes lay in the encoding sequence of ORI binding, helicase, and transactivation functional domains (Figure 2), despite only helicase domains being analyzed in great detail [23]. This region includes the convergent mutation between the CPV and FPLV clades (change Q to E in some FPLV strains) at the positively selected site 545 and the only residue subjected to positive selection pressure within the FPLV strains (site 443). Whereas residue 443 putatively lies in the β3-sheet of the Walker motif B of the helicase domain protein sequence, residues 350 and 544–545 are located between the α5- and α6-helices and just close to the α11-helix of the same domain, respectively, as illustrated in Canuti et al. [70] and Niskanen et al. [23]. The evidence of the convergent or divergent amino-acid changes between FPLV and CPV could contribute to further elucidate NS1 protein structure by clarifying any potential role of these residues. Moreover, the lack of studies on the *NS2* gene sequence [24] and its encoded protein highlights the need for additional investigations to advance any hypothesis on the effect of changes observed in the NS2 aa sequences.

The analyzed sequences clustered mainly according to the viral lineage, suggesting divergent evolution between FPLV and CPV also for the *NS1* gene, but also according to the geographical area and the year of sample collection, especially for the CPV NS1 sequences. Separate clades in the phylogenetic tree included the FPLV and the CPV strains, and, although these differences are due to few aa changes, the molecular divergence could be considered as a useful tool in outbreak tracing [31,34]. Residues 23, 165, and 443 allow clustering old FPLV from domestic cats and wild carnivores from North America and Japan [34], suggesting a potential common origin. Similarly, common aa changes were also observed in the *NS2* encoding sequence of the FPLV strains from the USA and Canada. Although higher genetic stability compared to CPV was supposed, these few molecular markers contributed to distinguishing the FPLV strains and, therefore, we could consider changes at these residues as potential synapomorphies.

The lack of geographic segregation within the phylogenetic tree suggests the wide distribution of viruses, possibly by trading or transport of animals, as well as by contaminated equipment. As previously observed, infected animals represent a potential way of transport of CPV for long distances [36], as also evidenced for other canine viruses [71,72,73], and further analyses are necessary to evaluate both the spread and evolution within the canine population of variants that reach a new location.

The comparison with the phylogenetic tree inferred from VP2 sequences shows that, based on this dataset, the CPV sequences did not form clades corresponding to the CPV variant (2a/2b/2c) and showed different phylogenetic relationships, possibly because of the different evolutive forces acting on the different genes. As hypothesized by other authors, the CPV antigenic variants (CPV-2a/2b/2c) could be considered as variants of CPV-2a rather than distinct subtypes [74] and the classification system based on a single amino acid (VP2 426) to identify CPV variants does not reflect phylogenetic relationships of the strains; thus, it is not suitable to analyze CPV evolution [75]. Indeed, the molecular characterization of both ORFs allows possibly reconsidering the current typing of CPV (2a/2b/2c), which is not phylogenetically supported. The aa changes in the *VP2* gene considered for viral typing probably arose independently in different countries, also considering the global spread of CPV through animal movements, and they are not reliable in defining the viral evolution. Therefore, the molecular analysis based on long genome sequences encompassing both major ORFs could be helpful in the epidemiological surveillance of both CPV and FPLV, supporting the tracking of viral spread, and could contribute to further elucidate the evolution of *Carnivore protoparvovirus 1*.

In conclusion, a continuous molecular survey is necessary to better elucidate the ecology and distribution of the described strains and to evaluate their fit in the canine and feline populations. The reductionist evaluations based only on the *VP2* genomic sequence should be replaced by a holistic molecular approach, based on analysis of both ORFs, and the description of new CPV and FPLV mutants should include at least the major structural and nonstructural proteins.

## Figures and Tables

**Table 1:** Origin and information on samples of cats and dogs. FPLV—feline panleukopenia virus; CPV—canine parvovirus.

Sample Id *	Date of Sampling	Origin	Sample	Type	Accession Number	Reference
72752/13	23 Oct 2013	Cat	Spleen	FPLV	MK413724 ^a^	This study
4311/14	21 Jan 2014	Cat	Spleen	FPLV	MK413725 ^a^	This study
149/15	05 Jan 2015	Cat	Intestine	FPLV	MK413726 ^a^	This study
**3201c1/15**	24 Nov 2015	Cat	Intestine	FPLV	KX434461 ^a^	This study
**38056c2/15**	26 Aug 2015	Cat	Intestine	FPLV	MK413727 ^a^	This study
32369/15	14 Jul 2015	Cat	Intestine	FPLV	MK413728 ^a^	This study
**42807/15**	24 Nov 2015	Cat	Rectal swab	FPLV	KX434462 ^a^	This study
52333eva/15	18 Nov 2015	Cat	Heart	FPLV	MK413729 ^a^	This study
**55611/15**	07 Dec 2015	Cat	Intestine	FPLV	MK413730 ^a^	This study
**58774/15**	23 Dec 2015	Cat	Intestine	FPLV	MK413731 ^a^	This study
**PA285c2/16**	12 Jan 2016	Cat	Intestine	FPLV	MK413732 ^b^	This study
RG21/16	04 Jan 2016	Cat	Spleen	FPLV	MK413733 ^b^	This study
PA12880Fe/16	11 Apr 2016	Cat	Intestine	FPLV	MK413734 ^b^	This study
PA12880Re/16	11 Apr 2016	Cat	Spleen	FPLV	MK413735 ^b^	This study
PA12880Mi/16	11 Apr 2016	Cat	Intestine	FPLV	MK413736 ^b^	This study
**PA11334/17**	20 Apr 2017	Cat	Brain	FPLV	MK413737 ^a^	This study
CT1375/17	20 Feb 2017	Cat	Spleen	FPLV	MK413738 ^a^	This study
**29451/09**	10 Sep 2009	Dog	Intestine	CPV-2a	KX434454 ^a^	This study
**987/10**	14 Jul 2010	Dog	Intestine	CPV-2a	KX434457 ^a^	This study
PA40697/16	02 Nov 2016	Dog	Spleen	CPV-2a	MK413739 ^b^	This study
**PA43847/16**	21 Nov 2016	Dog	Rectal swab	CPV-2a	MG434738 ^a^	[32]
**PA48686/16**	21 Dec 2016	Dog	Intestine	CPV-2a	MG434739 ^a^	[32]
**PA3213/17**	09 Feb 2017	Dog	Intestine	CPV-2a	MG434740 ^a^	[32]
**PA5610/17**	03 Mar 2017	Dog	Rectal swab	CPV-2a	MG434741 ^a^	[32]
**PA10388/17**	11 Apr 2017	Dog	Spleen	CPV-2a	MG434742 ^a^	[32]
**PA13577/17**	15 May 2017	Dog	Spleen	CPV-2a	MG434743 ^a^	[32]
**PA13579id90/17**	15 May 2017	Dog	Intestine	CPV-2a	MG434744 ^a^	[32]
**PA13579id93/17**	15 May 2017	Dog	Spleen	CPV-2a	MG434745 ^a^	[32]
PA30636/17	31 Oct 2017	Dog	Spleen	CPV-2a	MK413740 ^a^	This study
PA31209/17	07 Nov 2017	Dog	Spleen	CPV-2a	MK413741 ^a^	This study
PA13600/17	15 May 2017	Dog	Spleen	CPV-2b	MK413742 ^a^	This study
**23782/09**	10 Sep 2009	Dog	Intestine	CPV-2c	KX434455 ^a^	This study
**25835/09**	10 Sep 2009	Dog	Intestine	CPV-2c	KU508407 ^a^	This study
**45361/09**	21 Oct 2009	Dog	Intestine	CPV-2c	KX434456 ^a^	This study
**2323/11**	21 Jun 2011	Dog	Intestine	CPV-2c	KX434458 ^a^	This study
**27692c1/11**	05 Jul 2011	Dog	Intestine	CPV-2c	KX434459 ^a^	This study
**52238/12**	20 Oct 2012	Dog	Intestine	CPV-2c	KX434460 ^a^	This study
PA15423/16	29 Apr 2016	Cat	Spleen	CPV-2c	MK413743 ^a^	This study
PA36395/16	06 Oct 2016	Dog	Intestine	CPV-2c	MK413744 ^a^	This study
PA39667/16	26 Oct 2016	Dog	Brain	CPV-2c	MK413745 ^b^	This study
**41113c1/16**	03 Nov 2016	Dog	Rectal swab	CPV-2c	MF510158 ^a^	[36]
**PA41113c2/16**	03 Nov 2016	Dog	Rectal swab	CPV-2c	MK413746 ^a^	This study
**PA45984/16**	01 Dec 2016	Dog	Rectal swab	CPV-2c	MK413747 ^a^	This study
**2743/17**	06 Feb 2017	Dog	Intestine	CPV-2c	MF510157 ^a^	[36]
CT1839id0018/17	02 Mar 2017	Dog	Intestine	CPV-2c	MK413748 ^a^	This study
CT1839id2213/17	02 Mar 2017	Dog	Intestine	CPV-2c	MK413749 ^a^	This study
PA27184/17	29 Sep 2017	Dog	Rectal swab	CPV-2c	MK413750 ^a^	This study

* Samples in bold correspond to strain isolated. ^a^ ORF1 and ORF2 sequences; ^b^ *NS1* gene sequence.

**Table 2:** *NS1* non-synonymous changes of analyzed FPLV strains described in this study.

Strain	NS1 Amino Acids (Nucleotides) ^a^						
	81 (241–243)	115 (343–345)	247 (739–741)	248 (742–744)	545 (1633–1635)	595 (1783–1785)	664 (1990–1992)
72752/13	V (GTT)	I (ATT)	H (CAT)	T (ACT)	E (GAA)	H (CAC)	Q (CAA)
4311/14	--	--	--	--	--	--	--
149/15	I (ATT)	V (GTT)	Q (CAA)	--	--	Q (CAA)	R (CGA)
3201c1/15	--	V (GTT)	Q (CAA)	--	--	Q (CAA)	R (CGA)
55611/15	--	V (GTT)	Q (CAA)	--	--	Q (CAA)	R (CGA)
RG21/16	--	V (GTT)	Q (CAA)	--	--	Q (CAA)	R (CGA)
38056c/15	--	--	--	--	--	--	--
32369/15	--	--	--	--	--	--	--
42807/15	--	--	--	--	--	--	--
52333eva/15	--	--	--	--	--	--	--
58774/15	--	--	--	--	--	--	--
PA285c2/16	--	--	--	--	--	--	--
PA12880Felix/16	--	--	--	--	--	--	--
PA12880Red/16	--	--	--	--	--	--	--
PA12880Miele/16	--	--	--	--	--	--	--
PA11334/17	--	--	--	--	--	--	--
CT1375/17	--	--	--	--	--	--	--

^a^ Amino-acid and nucleotide (in brackets) positions refer to the prototype FPLV isolate FPV-4.us_64 (U.S.A.–1964; accession n.: EU659112). Sites where no variation was observed are marked by “--“.

**Table 3:** *NS1* non-synonymous changes of analyzed CPV strains described in this study.

Strain	NS1 Amino Acids (Nucleotides) ^b^														
	CPV Variant	60 (178–180)	239 (715–717)	247 (739–741)	248 (742–744)	350 (1048–1050)	397 (1189–1191)	544 (1630–1632)	545 (1633–1635)	572 (1714–1716)	584 (1750–1752)	590 (1768–1770)	595 (1783–1785)	597 (1789–1791)	630 (1888–1890)
29451/09	CPV-2a	I (ATT)	N (AAC)	Q (CAA)	I (ATT)	D (GAT)	L (CTT)	Y (TAT)	E (GAA)	K (AAA)	T (ACA)	P (CCT)	Q (CAA)	L (CTA)	L (CTT)
987/10	CPV-2a	--	--	--	--	N (AAT)	--	F (TTT)	--	E (GAA)	A (GCA)	--	--	--	--
PA40697/16	CPV-2a	--	--	--	--	N (AAT)	--	F (TTT)	--	E (GAA)	--	--	--	P (CCA)	--
PA43847/16	CPV-2a	--	T (ACC)	--	--	N (AAT)	--	F (TTT)	--	E (GAA)	--	--	--	P (CCA)	--
PA48686/16	CPV-2a	--	--	--	--	N (AAT)	--	F (TTT)	--	E (GAA)	--	--	--	P (CCA)	--
PA3213/17	CPV-2a	--	--	--	--	N (AAT)	F (TTT)	F (TTT)	--	E (GAA)	--	--	--	P (CCA)	--
PA5610/17	CPV-2a	--	--	--	--	N (AAT)	--	F (TTT)	--	E (GAA)	--	--	--	P (CCA)	--
PA10388/17	CPV-2a	--	--	--	--	N (AAT)	--	F (TTT)	--	E (GAA)	--	--	--	P (CCA)	--
PA13577/17	CPV-2a	--	--	--	--	N (AAT)	--	F (TTT)	--	E (GAA)	--	--	--	P (CCA)	--
PA13579id90/17	CPV-2a	--	--	--	--	N (AAT)	--	F (TTT)	--	E (GAA)	--	--	--	P (CCA)	--
PA13579id93/17	CPV-2a	--	--	--	--	N (AAT)	--	F (TTT)	--	E (GAA)	--	--	--	P (CCA)	--
PA30636/17	CPV-2a	--	--	--	--	N (AAT)	--	F (TTT)	--	E (GAA)	--	--	--	P (CCA)	--
PA31209/17	CPV-2a	--	--	--	--	N (AAT)	--	F (TTT)	--	E (GAA)	--	--	--	P (CCA)	--
PA13600/17	CPV-2b	--	--	--	--	-- (AAC)	--	--	--	--	--	S (TCT)	--	P (CCA)	--
23782/09	CPV-2c	--	--	--	--	--	--	--	--	E (GAA)	-- (ACG)	--	--	--	--
25835/09	CPV-2c	--	--	--	--	--	--	--	--	E (GAA)	-- (ACG)	--	--	--	--
45361/09	CPV-2c	--	--	--	--	--	--	--	--	E (GAA)	-- (ACG)	--	--	--	--
2323/11	CPV-2c	--	--	--	--	--	--	--	--	E (GAA)	-- (ACG)	--	--	--	--
27692c1/11	CPV-2c	--	--	--	--	--	--	--	--	E (GAA)	-- (ACG)	--	--	--	--
52238/12	CPV-2c	--	--	--	--	--	--	--	--	E (GAA)	-- (ACG)	--	--	--	--
PA15423/16	CPV-2c	--	--	--	--	--	--	--	--	E (GAA)	-- (ACG)	--	--	--	--
PA36395/16	CPV-2c	--	--	--	--	--	--	--	--	E (GAA)	-- (ACG)	--	--	--	--
PA39667/16	CPV-2c	--	--	--	--	--	--	--	--	E (GAA)	-- (ACG)	--	--	--	--
41113c1/16	CPV-2c	--	--	--	--	--	--	--	--	E (GAA)	-- (ACG)	--	--	--	--
PA41113c2/16	CPV-2c	--	--	--	--	--	--	--	--	E (GAA)	-- (ACG)	--	--	--	--
PA45984/16	CPV-2c	--	--	--	--	--	--	--	--	E (GAA)	-- (ACG)	--	--	--	--
2743/17	CPV-2c	V (GTT)	--	--	--	--	--	F (TTT)	V (GTA)	E (GAA)	--	--	--	--	P (CCT)
CT1839id0018/17	CPV-2c	--	--	--	--	--	--	--	--	E (GAA)	-- (ACG)	--	--	--	--
CT1839id2213/17	CPV-2c	--	--	--	--	--	--	--	--	E (GAA)	-- (ACG)	--	--	--	--
PA27184/17	CPV-2c	--	--	--	--	--	--	--	--	E (GAA)	-- (ACG)	--	--	--	--

^b^ Amino-acid and nucleotide (in brackets) positions refer to the prototype CPV strain CPV-N (U.S.A. – 1978; accession n.: M19296). Sites where no variation was observed are marked by “--“.

**Table 4:** List of codons within *NS1* gene sequence identified as being under negative or positive selection pressure. FUBAR—Fast Unconstrained Bayesian Approximation for inferring selection; MEME—Mixed-Effects Model of Evolution.

	Sites Under Negative Selection Pressure *	Sites Under Positive Selection Pressure	
	FUBAR	FUBAR	MEME
CPV	7, 10, 14, 15, **31**, 32, 47, 53, 54, 56, 66, 68, 69, 83, 92, 99, **102**, 104, 105, 107, 114, 119, 123, 124, 132, 135, 137, 140, 154, 163, 164, 165, 170, 172, 179, 189, 200, 211, 219, 223, 240, 242, 250, 251, 279, 283, **284**, 297, **307**, 313, **323**, 324, 325, 333, 336, 337, 340, 341, 343, 349, 353, 360, 366, 371, 374, 378, 384, 388, 391, 393, 394, 395, **403**, 405, 408, 430, 432, **435**, 439, 444, 451, 459, 463, 467, 473, 474, 475, 476, 483, **488**, **489**, 494, 495, 497, 499, 503, 505, 506, 512, 514, **517**, 525, 527, 528, 529, 531, 536, 537, **541**, **543**, 554, **560**, 563, 564, 584, 591, 596, 633, 640, 641, 642, 657, 659, 662	19, 278, 545, 572, 583	278, 572, 583, 597, 647
FPLV	6, **31**, 39, 58, 60, 71, 97, **102**, 174, 177, 185, 201, 207, 270, **284**, **307**, **323**, 352, 357, **403**, 418, 422, 428, **435**, 462, 479, **488**, **489**, 493, 515, **517**, 520, 533, 540, **541**, **543**, 549, 551, **560**, 562, 653, 660	443	

* codons in bold correspond to those identified in both CPV and FPV lineages.

## Supplementary Materials

The following are available online at https://www.mdpi.com/1999-4915/11/4/308/s1: Dataset S1: Amino-acid sequence variations in the *NS1* gene sequence of CPV/FPLV and of the reference viruses from the NCBI database. Table S2: VP2 non-synonymous changes of analyzed FPLV/CPV strains described in this study. Dataset S3: Amino-acid sequence variations in the *NS2* gene sequence of CPV/FPLV collected in Italy. Recombination and Selection Pressure S4: Recombination and selection pressure file. Recombination S5: Phylogenetic reconstructions of the complete *NS1* gene of CPV-2 and FPLV strains based on different genomic regions located upstream and downstream the putative recombination breakpoint (nt 1515). Figure S6: Maximum-likelihood tree based on 133 complete *NS1* gene sequences of feline panleukopenia virus (FPLV, in green) and canine parvovirus type-2 (CPV-2, in black) strains (bootstrap 1000 replicates; bootstrap values shown greater than 65). Green triangles and black dots indicate, respectively, FPLV and CPV strains analyzed in this study. Each sequence is indicated with virus type (FPLV/CPV) or variant (CPV-2, CPV-2a, CPV-2b, CPV-2c), country and year of collection, strain/isolate name, and accession number. The term “new” was used to distinguish the CPV-2a/2b strains with S297A VP2 amino-acid change from the early CPV-2a/2b variants. Figure S7: Maximum-likelihood tree based on 132 complete *VP2* gene sequences of feline panleukopenia virus (FPLV, in green) and canine parvovirus type-2 (CPV-2, in black) strains (bootstrap 1000 replicates; bootstrap values greater than 65 are shown). Green triangles and black dots indicate, respectively, FPLV and CPV strains analyzed in this study. Each sequence is indicated with virus type (FPLV/CPV) or variant (CPV-2, CPV-2a, CPV-2b, CPV-2c), country and year of collection, strain/isolate name, and accession number. The term “new” was used to distinguish the CPV-2a/2b strains with S297A VP2 amino-acid change from the early CPV-2a/2b variants.
