# Supplementary material for: Molecular Characterization and Evolutionary Analyses of Carnivore Protoparvovirus 1 NS1 Gene
Source: Viruses. 2019 Mar 29;11(4):308. doi: 10.3390/v11040308 (PMC6520740; doi:10.3390/v11040308)
Supplement: Supplementary file 1 [file viruses-11-00308-s001.zip › Supplementary table S2.docx]

**Supplementary table S2.** *VP2* non-synonymous changes of analyzed FPLV strains described in this study.

| **Strain** | **VP2 Amino Acids (nucleotides)^a^** | | |
| --- | --- | --- | --- |
|  | **232**  **(694-696)** | **311**  **(931-933)** | **359**  **(1075-1077)** |
| 72752/13 | I  (ATA) | N  (AAT) | G  (GGA) |
| 4311/14 | -- | -- | -- |
| 149/15 | V  (GTA) | D  (GAT) | -- |
| 3201c1/15 | V  (GTA) | D  (GAT) | A  (GCA) |
| 55611/15 | V  (GTA) | D  (GAT) | A  (GCA) |
| RG21/16 | V  (GTA) | D  (GAT) | A  (GCA) |
| 38056c2/15 | -- | -- | -- |
| 32369/15 | -- | D  (GAT) | -- |
| 42807/15 | -- | -- | -- |
| 52333eva/15 | -- | D  (GAT) | -- |
| 58774/15 | -- | D  (GAT) | -- |
| PA285c2/16 | -- | D  (GAT) | -- |
| PA12880Fe/16 | -- | D  (GAT) | -- |
| PA12880Re/16 | -- | D  (GAT) | -- |
| PA12880Mi/16 | -- | D  (GAT) | -- |
| PA11334/17 | -- | D  (GAT) | -- |
| CT1375/17 | -- | -- | -- |

^a^Amino acid and nucleotide (in brackets) positions are referred to the prototype FPLV isolate FPV-4.us_64 (U.S.A. – 1964; accession n.: EU659112). Sites where no variations was observed are marked by “—“.

**Supplementary table S2.** *VP2* non-synonymous changes of analyzed CPV strains described in this study.

| **Strain** |  | **NS1 Amino Acids ( Nucleotides)^b^** | | | | | | | | |
| --- | --- | --- | --- | --- | --- | --- | --- | --- | --- | --- |
|  | **CPV**  **Variant** | **5**  **(13-15)** | **13**  **(37-39)** | **139**  **(415-417)** | **267**  **(799-801)** | **324**  **(970-972)** | **370**  **(1108-1110)** | **371**  **(1111-1113)** | **418**  **(1252-1254)** | **426**  **(1276-1278)** |
| 29451/09 | CPV-2a | A  (GCA) | P  (CCT) | V  (GTT) | F  (TTT) | Y  (TAT) | Q  (CAA) | A  (GCA) | I  (ATT) | N  (AAT) |
| 987/10 | CPV-2a | -- | -- | -- | -- | -- | -- | -- | -- | -- |
| PA40697/16 | CPV-2a | -- | -- | -- | -- | L  (CTT) | -- | -- | -- | -- |
| PA43847/16 | CPV-2a | -- | -- | -- | -- | L  (CTT) | -- | -- | -- | -- |
| PA48686/16 | CPV-2a | -- | -- | -- | -- | L  (CTT) | -- | -- | -- | -- |
| PA3213/17 | CPV-2a | -- | -- | -- | -- | L  (CTT) | -- | -- | -- | -- |
| PA5610/17 | CPV-2a | -- | -- | -- | -- | L  (CTT) | -- | -- | -- | -- |
| PA10388/17 | CPV-2a | -- | -- | -- | -- | L  (CTT) | -- | -- | -- | -- |
| PA13577/17 | CPV-2a | -- | -- | -- | -- | L  (CTT) | -- | -- | -- | -- |
| PA13579id90/2017 | CPV-2a | -- | -- | -- | -- | L  (CTT) | -- | -- | -- | -- |
| PA13579id93/2017 | CPV-2a | -- | -- | -- | -- | L  (CTT) | -- | -- | -- | -- |
| PA30636/17 | CPV-2a | -- | -- | -- | -- | L  (CTT) | -- | -- | -- | -- |
| PA31209/17 | CPV-2a | -- | -- | -- | -- | L  (CTT) | -- | -- | -- | -- |
| PA13600/17 | CPV-2b | -- | S  (TCT) | -- | -- | -- | -- | G  (GGA) | T  (ACT) | D  (GAT) |
| 23782_09 | CPV-2c | -- | -- | -- | -- | -- | -- | -- | -- | E  (GAA) |
| 25835_09 | CPV-2c | -- | -- | -- | -- | -- | -- | -- | -- | E  (GAA) |
| 45361_09 | CPV-2c | -- | -- | -- | -- | -- | -- | -- | -- | E  (GAA) |
| 2323_11 | CPV-2c | -- | -- | -- | -- | -- | -- | -- | -- | E  (GAA) |
| 27692c1/11 | CPV-2c | -- | -- | -- | -- | -- | -- | -- | -- | E  (GAA) |
| 52238/12 | CPV-2c | -- | -- | -- | -- | -- | -- | -- | -- | E  (GAA) |
| PA15423/16 | CPV-2c | -- | -- | I  (ATT) | -- | -- | -- | -- | -- | E  (GAA) |
| PA36395/16 | CPV-2c | -- | -- | I  (ATT) | -- | -- | -- | -- | -- | E  (GAA) |
| PA39667/16 | CPV-2c | -- | -- | I  (ATT) | -- | -- | -- | -- | -- | E  (GAA) |
| 41113c1/16 | CPV-2c | -- | -- | I  (ATT) | -- | -- | -- | -- | -- | E  (GAA) |
| PA41113c2/16 | CPV-2c | -- | -- | I  (ATT) | -- | -- | -- | -- | -- | E  (GAA) |
| PA45984/16 | CPV-2c | -- | -- | -- | -- | -- | -- | -- | -- | E  (GAA) |
| 2743/17 | CPV-2c | G  (GGA) | -- | -- | Y  (TAT) | I  (ATT) | R  (CGA) | -- | -- | E  (GAA) |
| CT1839id0018/17 | CPV-2c | -- | -- | -- | -- | -- | -- | -- | -- | E  (GAA) |
| CT1839id2213/17 | CPV-2c | -- | -- | -- | -- | -- | -- | -- | -- | E  (GAA) |
| PA27184/17 | CPV-2c | -- | -- | -- | -- | -- | -- | -- | -- | E  (GAA) |

^b^Amino acid and nucleotide (in brackets) positions are referred to the prototype CPV strain CPV-N (U.S.A. – 1978; accession n.: M19296). Sites where no variation was observed are marked by “—“.
