# Supplementary material for: Molecular Characterization and Evolutionary Analyses of Carnivore Protoparvovirus 1 NS1 Gene
Source: Viruses. 2019 Mar 29;11(4):308. doi: 10.3390/v11040308 (PMC6520740; doi:10.3390/v11040308)
Supplement: Supplementary file 1 [file viruses-11-00308-s001.zip › Dataset S1.docx]

**Dataset 1.** Amino acid sequence variations in the NS1 gene sequence of CPV/FPLV and of the reference viruses from the NCBI database.

|  |  |  |  | **NS1 amino acid residues** | | | | | | | | | | |
| --- | --- | --- | --- | --- | --- | --- | --- | --- | --- | --- | --- | --- | --- | --- |
| **Type/Variant** | **Country** | **Year** | **Acc.n.** | **23** | **115** | **165** | **247** | **350** | **443** | **544** | **545** | **595** | **597** | **664** |
| FPLV | USA | 1967 | M38246 | D | V | V | H | D | V | Y | Q | H | L | R |
| FPLV | Italy | 2015 | KX434462 | N | I |  |  |  | I |  | E |  |  | Q |
| FPLV | Italy | 2015 | KX434461 | N |  |  | Q |  | I |  | E | Q |  |  |
| FPLV | Italy | 2016 | MK413732 | N | I |  |  |  | I |  | E |  |  | Q |
| FPLV | Italy | 2016 | MK413734 | N | I |  |  |  | I |  | E |  |  | Q |
| FPLV | Italy | 2016 | MK413736 | N | I |  |  |  | I |  | E |  |  | Q |
| FPLV | Italy | 2016 | MK413735 | N | I |  |  |  | I |  | E |  |  | Q |
| FPLV | Italy | 2015 | MK413727 | N | I |  |  |  | I |  | E |  |  | Q |
| FPLV | Italy | 2015 | MK413729 | N | I |  |  |  | I |  | E |  |  | Q |
| FPLV | Italy | 2015 | MK413730 | N |  |  | Q |  | I |  | E | Q |  |  |
| FPLV | Italy | 2015 | MK413731 | N | I |  |  |  | I |  | E |  |  | Q |
| FPLV | Italy | 2017 | MK413737 | N | I |  |  |  | I |  | E |  |  | Q |
| FPLV | Italy | 2016 | MK413733 | N |  |  | Q |  | I |  | E | Q |  |  |
| FPLV | Italy | 2017 | MK413738 | N | I |  |  |  | I |  | E |  |  | Q |
| FPLV | Italy | 2015 | MK413728 | N | I |  |  |  | I |  | E |  |  | Q |
| FPLV | Italy | 2013 | MK413724 | N | I |  |  |  | I |  | E |  |  | Q |
| FPLV | Italy | 2015 | MK413726 | N |  |  | Q |  | I |  | E | Q |  |  |
| FPLV | Italy | 2014 | MK413725 | N | I |  |  |  | I |  | E |  |  | Q |
| FPLV | USA | 1964 | EU659112 | N |  |  |  |  | I |  |  |  |  |  |
| FPLV | USA | 1967 | EU659111 |  |  |  |  |  |  |  |  |  |  |  |
| FPLV | USA | 1967 | M38246 |  |  |  |  |  |  |  |  |  |  |  |
| FPLV | France | 1968 | AB000057 | N |  |  |  |  |  |  |  |  |  |  |
| FPLV | Australia | 1970 | X55115 | N |  |  |  |  | I |  |  |  |  |  |
| FPLV | Japan | 1974 | AB000055 | N |  |  |  |  | I |  |  |  |  |  |
| FPLV | Japan | 1975 | AB000065 | N |  |  |  |  | I |  |  |  |  |  |
| FPLV | Japan | 1976 | AB000069 | N |  |  |  |  | I |  |  |  |  |  |
| FPLV | Japan | 1979 | AB000062 | N |  |  |  |  |  |  |  |  |  |  |
| FPLV | Japan | 1979 | AB000063 | N |  |  |  |  | I |  |  |  |  |  |
| FPLV | China | 1986 | KX900570 | N |  |  | Q |  | I |  | E | Q |  |  |
| FPLV | USA | 1989 | EU659114 |  |  | I |  |  |  |  |  |  |  |  |
| FPLV | USA | 1989 | EU659113 |  |  | I |  |  |  |  |  |  |  |  |
| FPLV | Japan | 1990 | AB000048 |  |  | I |  |  |  |  |  |  |  |  |
| FPLV | Japan | 1993 | AB000053 | N |  |  |  |  | I |  |  |  |  |  |
| FPLV | Japan | 1994 | AB000051 | N |  |  |  |  | I |  |  |  |  |  |
| FPLV | Japan | 1994 | AB000049 |  |  | I |  |  |  |  |  |  |  |  |
| FPLV | Japan | 1995 | AB000060 |  |  | I |  |  |  |  |  |  |  |  |
| FPLV | USA | 2006 | EU659115 |  |  |  |  |  |  |  |  |  |  |  |
| FPLV | China | 2007 | EF988660 | N |  |  | Q |  | I |  | E | Q |  |  |
| FPLV | Canada | 2010 | MF069446 | N |  |  |  |  |  |  |  |  |  |  |
| FPLV | China | 2014 | KP280068 | N |  |  | Q |  | I |  | E | Q |  |  |
| FPLV | Belgium | 2013 | KP769859 | N |  |  | Q |  | I |  | E | Q |  |  |
| FPLV | Canada | 2015 | MF069445 |  |  | I |  |  |  |  |  |  |  |  |
| FPLV | Canada | 2016 | MF069447 |  |  | I |  |  |  |  |  |  |  |  |
| FPLV | China | 2016 | KX685354 | N |  |  | Q |  | I |  | E | Q |  |  |
|  |  |  |  |  |  |  |  |  |  |  |  |  |  |  |
| **Type/Variant^a^** | **Country** | **Year** | **Acc.n.** | **23** | **115** | **165** | **247** | **350** | **443** | **544** | **545** | **595** | **597** | **664** |
| CPV-2 | USA | 1978 | M19296 | N | V | V | Q | D | I | Y | E | Q | L | R |
| new CPV-2a | Italy | 2009 | KX434454 |  |  |  |  |  |  |  |  |  |  |  |
| new CPV-2a | Italy | 2010 | KX434457 |  |  |  |  | N |  | F |  |  |  |  |
| new CPV-2a | Italy | 2017 | MK413740 |  |  |  |  | N |  | F |  |  | P |  |
| new CPV-2a | Italy | 2017 | MK413741 |  |  |  |  | N |  | F |  |  | P |  |
| new CPV-2a | Italy | 2016 | MK413739 |  |  |  |  | N |  | F |  |  | P |  |
| new CPV-2a | Italy | 2017 | MG434738 |  |  |  |  | N |  | F |  |  | P |  |
| new CPV-2a | Italy | 2016 | MG434739 |  |  |  |  | N |  | F |  |  | P |  |
| new CPV-2a | Italy | 2017 | MG434740 |  |  |  |  | N |  | F |  |  | P |  |
| new CPV-2a | Italy | 2017 | MG434741 |  |  |  |  | N |  | F |  |  | P |  |
| new CPV-2a | Italy | 2017 | MG434742 |  |  |  |  | N |  | F |  |  | P |  |
| new CPV-2a | Italy | 2017 | MG434743 |  |  |  |  | N |  | F |  |  | P |  |
| new CPV-2a | Italy | 2017 | MG434744 |  |  |  |  | N |  | F |  |  | P |  |
| **Type/Variant^a^** | **Country** | **Year** | **Acc.n.** | **23** | **115** | **165** | **247** | **350** | **443** | **544** | **545** | **595** | **597** | **664** |
| new CPV-2a | Italy | 2017 | MG434745 |  |  |  |  | N |  | F |  |  | P |  |
| CPV-2b | Italy | 2017 | MK413742 |  |  |  |  |  |  | F |  |  | P |  |
| CPV-2c | Italy | 2009 | KX434455 |  |  |  |  |  |  |  |  |  |  |  |
| CPV-2c | Italy | 2009 | KX434456 |  |  |  |  |  |  |  |  |  |  |  |
| CPV-2c | Italy | 2009 | KU508407 |  |  |  |  |  |  |  |  |  |  |  |
| CPV-2c | Italy | 2011 | KX434458 |  |  |  |  |  |  |  |  |  |  |  |
| CPV-2c | Italy | 2011 | KX434459 |  |  |  |  |  |  |  |  |  |  |  |
| CPV-2c | Italy | 2012 | KX434460 |  |  |  |  |  |  |  |  |  |  |  |
| CPV-2c | Italy | 2016 | MK413743 |  |  |  |  |  |  |  |  |  |  |  |
| CPV-2c | Italy | 2016 | MK413745 |  |  |  |  |  |  |  |  |  |  |  |
| CPV-2c | Italy | 2016 | MF510158 |  |  |  |  |  |  |  |  |  |  |  |
| CPV-2c | Italy | 2016 | MK413747 |  |  |  |  |  |  |  |  |  |  |  |
| CPV-2c | Italy | 2016 | MK413744 |  |  |  |  |  |  |  |  |  |  |  |
| CPV-2c | Italy | 2016 | MK413746 |  |  |  |  |  |  |  |  |  |  |  |
| CPV-2c | Italy | 2017 | MF510157 |  |  |  |  |  |  | F | V |  |  |  |
| CPV-2c | Italy | 2017 | MK413748 |  |  |  |  |  |  |  |  |  |  |  |
| CPV-2c | Italy | 2017 | MK413749 |  |  |  |  |  |  |  |  |  |  |  |
| CPV-2c | Italy | 2017 | MK413750 |  |  |  |  |  |  |  |  |  |  |  |
| new CPV-2b | Germany | 1995 | AY742934 |  |  |  |  |  |  |  |  |  |  |  |
| CPV-2a | USA | 1981 | EU659118 |  |  |  |  |  |  |  |  |  |  |  |
| CPV-2 | USA | 1978 | EU659116 |  |  |  |  |  |  |  |  |  |  |  |
| CPV-2a | USA | 1984 | AY787926 |  |  |  |  |  |  |  |  |  |  |  |
| CPV | -- | -- | M38245 |  |  |  |  |  |  |  |  |  |  |  |
| CPV-2 | USA | 1978 | M19296 |  |  |  |  |  |  |  |  |  |  |  |
| CPV-2b | USA | 1990 | AY787928 |  |  |  |  |  |  | F |  |  |  |  |
| new CPV-2b | USA | 1998 | EU659120 |  |  |  |  |  |  |  |  |  |  |  |
| CPV-2b | USA | 2000 | EU659119 |  |  |  |  |  |  |  |  |  |  |  |
| new CPV-2b | USA | 2003 | AY742952 |  |  |  |  |  |  |  |  |  |  |  |
| new CPV-2b | USA | 1991 | AY742932 |  |  |  |  |  |  |  |  |  |  |  |
| new CPV-2a | China | 2011 | JX660690 |  |  |  |  |  |  |  |  |  |  |  |
| CPV-2a | USA | 2010 | JN867618 |  |  |  |  |  |  | F |  |  |  |  |
| new CPV-2a | USA | 2009 | JN867612 |  |  |  |  |  |  |  |  |  |  |  |
| new CPV-2a | China | 2004 | EF011664 |  |  |  |  |  |  |  |  |  |  |  |
| CPV-2a | USA | 1983 | AY787929 |  |  |  |  | N |  | F |  |  |  |  |
| new CPV-2a | USA | 2003 | AY742954 |  |  |  |  |  |  |  |  |  |  |  |
| new CPV-2b | USA | 1999 | AY742950 |  |  |  |  |  |  |  |  |  |  |  |
| new CPV-2a | New Zealand | 1994 | AY742933 |  |  |  |  |  |  | F |  |  |  |  |
| new CPV-2a | China | 2011 | JQ268283 |  |  |  |  |  |  |  |  |  |  |  |
| new CPV-2a | Germany | 1995 | AY742935 |  |  |  |  | N |  | F |  |  |  |  |
| new CPV-2a | Vietnam | 2012 | LC214970 |  | I |  |  |  |  |  |  |  |  |  |
| new CPV-2b | Japan | 2017 | LC270891 |  |  |  |  |  |  |  |  |  |  |  |
| CPV-2c | Uruguay | 2011 | KM457142 |  |  |  |  |  |  |  |  |  |  |  |
| CPV-2c | Uruguay | 2007 | KM457109 |  |  |  |  |  |  |  |  |  |  |  |
| CPV-2c | Uruguay | 2007 | KM457107 |  |  |  |  |  |  |  |  |  |  |  |
| CPV-2c | Uruguay | 2006 | KM457106 |  |  |  |  |  |  |  |  |  |  |  |
| new CPV-2a | USA | 2009 | JN867614 |  |  |  |  |  |  | F |  |  |  |  |
| new CPV-2a | USA | 2009 | JN867613 |  |  |  |  |  |  |  |  |  |  |  |
| new CPV-2a | USA | 2007 | JN867610 |  |  |  |  |  |  |  |  |  |  |  |
| new CPV-2b | USA | 1998 | EU659121 |  |  |  |  |  |  |  |  |  |  |  |
| CPV-2b | USA | 1990 | AY787927 |  |  |  |  |  |  | F |  |  |  |  |
| new CPV-2b | USA | 1998 | AY742948 |  |  |  |  | N |  | F |  |  | P |  |
| new CPV-2a | China | 2017 | MF134808 |  |  |  |  |  |  |  |  |  |  |  |
| new CPV-2a | China | 2013 | KR002794 |  |  |  |  |  |  |  |  |  |  |  |
| CPV-2c | Uruguay | 2011 | KM457128 |  |  |  |  |  |  |  |  |  |  |  |
| CPV-2c | Uruguay | 2010 | KM457123 |  |  |  |  |  |  |  |  |  |  |  |
| CPV-2c | Uruguay | 2010 | KM457121 |  |  |  |  |  |  |  |  |  |  |  |
| CPV-2c | Uruguay | 2009 | KM457116 |  |  |  |  |  |  |  |  |  |  |  |
| CPV-2c | Uruguay | 2008 | KM457114 |  |  |  |  |  |  |  |  |  |  |  |
| CPV-2c | Uruguay | 2008 | KM457113 |  |  |  |  |  |  |  |  |  |  |  |
| new CPV-2a | China | 2010 | KF638400 |  |  |  |  |  |  |  |  |  |  |  |
| new CPV-2a | China | 2006 | EU310373 |  |  |  |  |  |  |  |  |  | H |  |
| new CPV-2b | USA | 2004 | AY742956 |  |  |  |  | N |  | F |  |  | P |  |
| CPV-2a | Japan | 1982 | D26079 |  |  |  |  |  |  | F |  |  |  |  |
| **Type/Variant^a^** | **Country** | **Year** | **Acc.n.** | **23** | **115** | **165** | **247** | **350** | **443** | **544** | **545** | **595** | **597** | **664** |
| new CPV-2a | Canada | 2016 | MF069444 |  |  |  |  |  |  |  |  |  |  |  |
| new CPV-2a | Canada | 2016 | MF069443 |  |  |  |  |  |  |  |  |  |  |  |
| new CPV-2a | China | 2014 | KR002802 |  |  |  |  |  |  |  |  |  |  |  |
| new CPV-2a | China | 2014 | KR002801 |  |  |  |  |  |  |  |  |  |  |  |
| new CPV-2a | China | 2013 | KR002798 |  |  |  |  |  |  |  |  |  |  |  |
| new CPV-2a | China | 2013 | KR002797 |  |  |  |  |  |  |  |  |  |  |  |
| new CPV-2a | China | 2014 | KP749864 |  |  |  |  |  |  |  |  |  |  |  |
| new CPV-2a | China | 2014 | KP749856 |  |  |  |  |  |  |  |  |  |  | Q |
| new CPV-2a | Uruguay | 2011 | KM457139 |  |  |  |  |  |  |  |  |  |  |  |
| new CPV-2a | Uruguay | 2010 | KM457133 |  |  |  |  |  |  |  |  |  |  |  |
| CPV-2c | Uruguay | 2011 | KM457131 |  |  |  |  |  |  |  |  |  |  |  |
| CPV-2c | Uruguay | 2011 | KM457130 |  |  |  |  |  |  |  |  |  |  |  |
| CPV-2c | Uruguay | 2011 | KM457127 |  |  |  |  |  |  |  |  |  |  |  |
| CPV-2c | Uruguay | 2010 | KM457126 |  |  |  |  |  |  |  |  |  |  |  |
| CPV-2c | Uruguay | 2010 | KM457120 |  |  |  |  |  |  |  |  |  |  |  |
| CPV-2c | Uruguay | 2009 | KM457115 |  |  |  |  |  |  |  |  |  |  |  |
| CPV-2c | Uruguay | 2007 | KM457110 |  |  |  |  |  |  |  |  |  |  |  |
| CPV-2c | Uruguay | 2006 | KM457105 |  |  |  |  |  |  |  |  |  |  |  |
| CPV-2c | Uruguay | 2006 | KM457104 |  |  |  |  |  |  |  |  |  |  |  |
| CPV-2c | Uruguay | 2006 | KM457103 |  |  |  |  |  |  |  |  |  |  |  |
| new CPV-2a | China | 2010 | HQ883272 |  |  |  |  |  |  |  |  |  |  |  |
| new CPV-2b | USA | 1998 | AY742946 |  |  |  |  | N |  | F |  |  | P |  |
| CPV-2c | Uruguay | 2009 | KM457118 |  |  |  |  |  |  |  |  |  |  |  |
| new CPV-2a | Canada | 2010 | MF069442 |  |  |  |  |  |  |  |  |  |  |  |
| new CPV-2a | Canada | 2014 | MF423125 |  |  |  |  |  |  |  |  |  |  |  |
| new CPV-2b | Japan | 2017 | LC270892 |  |  |  |  |  |  |  |  |  |  |  |
| new CPV-2a | Singapore | 2016 | KX618915 |  |  |  |  |  |  |  |  |  |  |  |
| new CPV-2a | China | 2014 | KR002804 |  |  |  |  |  |  |  |  |  |  |  |
| new CPV-2a | Uruguay | 2011 | KM457143 |  |  |  |  |  |  |  |  |  |  |  |
| new CPV-2a | Uruguay | 2011 | KM457135 |  |  |  |  |  |  |  |  |  |  |  |
| CPV-2c | Uruguay | 2011 | KM457129 |  |  |  |  |  |  |  |  |  |  |  |
| CPV-2c | Uruguay | 2011 | KM457124 |  |  |  |  |  |  |  |  |  |  |  |
| CPV-2c | Uruguay | 2009 | KM457117 |  |  |  |  |  |  |  |  |  |  |  |
| CPV-2c | Uruguay | 2008 | KM457111 |  |  |  |  |  |  |  |  |  |  |  |
| new CPV-2a | Uruguay | 2010 | KM457102 |  |  |  |  |  |  |  |  |  |  |  |
| new CPV-2a | USA | 2009 | JN867611 |  |  |  |  |  |  |  |  |  |  |  |
| new CPV-2b | Brazil | 2015 | KX774252 |  |  |  |  |  |  |  |  |  | P |  |
| new CPV-2a | China | 2013 | KR002795 |  |  |  |  |  |  |  |  |  |  |  |
| new CPV-2a | India | 2013 | KF366250 |  |  |  |  |  |  |  |  |  |  |  |
| new CPV-2a | Uruguay | 2011 | KM457140 |  |  |  |  |  |  |  |  |  |  |  |
| CPV-2c | Uruguay | 2011 | KM457125 |  |  |  |  |  |  |  |  |  |  |  |
| CPV-2c | Uruguay | 2010 | KM457122 |  |  |  |  |  |  |  |  |  |  |  |
| CPV-2c | Uruguay | 2010 | KM457119 |  |  |  |  |  |  |  |  |  | P |  |
| CPV-2c | Uruguay | 2007 | KM457108 |  |  |  |  |  |  |  |  |  |  |  |
| new CPV-2b | China | 2011 | JQ268284 |  |  |  |  |  |  |  | V |  |  |  |
| new CPV-2b | USA | 1998 | AY742936 |  |  |  |  |  |  |  |  |  |  |  |
| new CPV-2a | China | 2014 | KP749861 |  | I |  |  |  |  |  |  |  |  |  |
| new CPV-2a | USA | 2008 | JN867616 |  |  |  |  | N |  | F |  |  |  |  |
| new CPV-2a | USA | 2009 | JN867615 |  |  |  |  | N |  | F |  |  |  |  |
| new CPV-2b | Canada | 2014 | MF423123 |  |  |  |  |  |  | F |  |  | P |  |
| CPV-2c | Vietnam | 2013 | LC214969 |  |  |  |  |  |  |  |  |  |  |  |
| CPV-2c | Brazil | 2015 | KY073269 |  |  |  |  |  |  |  |  |  |  |  |
| CPV-2c | Australia | 2015 | KU508693 |  |  |  |  |  |  |  |  |  |  |  |
| CPV-2c | Australia | 2015 | KU508692 |  |  |  |  |  |  |  |  |  |  |  |
| CPV-2c | Australia | 2015 | KU508691 |  |  |  |  |  |  |  |  |  |  |  |
| new CPV-2a | China | 2014 | KR002800 |  |  |  |  |  |  | F | V |  |  |  |
| CPV-2c | Uruguay | 2008 | KM457112 |  |  |  |  |  |  |  |  |  |  |  |
| new CPV-2a | USA | 2008 | JN867617 |  |  |  |  | N |  | F |  |  |  |  |
| new CPV-2a | India |  | AJ564427 |  |  |  |  |  |  | F |  |  |  |  |
| new CPV-2b | Canada | 2014 | MF423124 |  |  |  |  |  |  | F |  |  | P |  |
| new CPV-2a | China | 2014 | KT382542 |  |  |  |  |  |  |  |  |  |  |  |
| new CPV-2a | China | 2013 | KR002792 |  |  |  |  |  |  | F | V |  |  |  |
| new CPV-2a | China | 2014 | KM014813 |  |  |  |  |  |  |  |  |  |  |  |
| **Type/Variant^a^** | **Country** | **Year** | **Acc.n.** | **23** | **115** | **165** | **247** | **350** | **443** | **544** | **545** | **595** | **597** | **664** |
| new CPV-2a | China | 2014 | KR002805 |  |  |  |  |  |  | F | V |  |  |  |
| new CPV-2a | China | 2014 | KR002803 |  |  |  |  |  |  |  |  |  |  |  |
| new CPV-2b | China | 2013 | KR002799 |  |  |  |  |  |  | F | V |  |  |  |
| new CPV-2b | China | 2013 | KR002793 |  |  |  |  |  |  | F | V |  |  |  |
| new CPV-2a | China | 2014 | KP749872 |  |  |  |  |  |  |  |  |  |  |  |
| new CPV-2a | China | 2014 | KP749868 |  |  |  |  |  |  | F | V |  |  |  |
| new CPV-2a | China | 2014 | KP749858 |  |  |  |  |  |  | F | V |  |  |  |
| new CPV-2a | China | 2009 | KF676668 |  |  |  |  |  |  | F | V |  |  |  |
| new CPV-2a | China | 2011 | JQ686671 |  |  |  |  |  |  | F | V |  |  |  |
| new CPV-2a | China | 2010 | HQ883270 |  |  |  |  |  |  | F | V |  |  |  |
| new CPV-2a | China | 2010 | HQ883266 |  |  |  |  |  |  | F | V |  |  |  |
| -- | India | -- | GQ421597 |  |  |  |  |  |  | F |  |  |  |  |
| new CPV-2a | China | 2008 | KY403998 |  |  |  |  |  |  | F | V |  |  |  |
| new CPV-2b | China | 2013 | KR002796 |  |  |  |  |  |  | F | V |  |  |  |
| new CPV-2b | China | 2014 | KP749874 |  |  |  |  |  |  |  |  |  |  |  |
| CPV-2c | China | 2014 | KP749870 |  |  |  |  |  |  | F | V |  |  |  |
| new CPV-2a | China | 2014 | KP749869 |  |  |  |  |  |  |  |  |  |  |  |
| new CPV-2a | China | 2010 | HQ883268 |  |  |  |  |  |  | F | V |  |  |  |
| new CPV-2b | China | 2014 | KP749866 |  |  |  |  |  |  |  |  |  |  |  |
| new CPV-2a | China | 2014 | KP749862 |  |  |  |  |  |  |  |  |  |  |  |
| new CPV-2b | China | 2014 | KP749871 |  |  |  |  |  |  |  |  |  |  |  |
| new CPV-2a | China | 2014 | KP749865 |  |  |  |  |  |  |  |  |  |  |  |
| new CPV-2a | China | 2014 | KP749857 |  |  |  |  |  |  |  |  |  |  |  |
| new CPV-2b | Brazil | 2015 | KX774251 |  |  |  |  |  |  | F |  |  |  |  |
| new CPV-2b | Brazil | 2014 | KX774249 |  |  |  |  |  |  | F |  |  |  |  |
| CPV-2c | China | 2014 | KP749873 |  |  |  |  |  |  |  |  |  |  |  |
| new CPV-2a | China | 2014 | KP749863 |  |  |  |  |  |  |  |  |  |  |  |
| new CPV-2a | China | 2014 | KP749860 |  |  |  |  |  |  | F | V |  |  |  |
| new CPV-2b | Brazil | 2016 | KX774250 |  |  |  |  |  |  | F |  |  |  |  |
| new CPV-2a | China | 2015 | MG583676 |  |  |  |  |  |  | F | V |  |  |  |
| new CPV-2a | China | 2014 | KP749867 |  |  |  |  |  |  | F | V |  |  |  |
| new CPV-2b | China | 2014 | KP749859 |  |  |  |  |  |  | F | V |  |  |  |
| new CPV-2b | Russia | 1993 | JN033694 |  |  |  | H |  |  | F | Q |  |  |  |

^a^The term “new” was used to distinguish the CPV-2a/2b strains with S297A VP2 amino acid change from the early CPV-2a/2b variants.
