# Supplementary material for: Molecular Characterization and Evolutionary Analyses of Carnivore Protoparvovirus 1 NS1 Gene
Source: Viruses. 2019 Mar 29;11(4):308. doi: 10.3390/v11040308 (PMC6520740; doi:10.3390/v11040308)
Supplement: Supplementary file 1 [file viruses-11-00308-s001.zip › Dataset S3.docx]

**Dataset 3.** Amino acid sequence variations in the NS2 gene sequence of CPV/FPLV collected in Italy.

|  |  |  | |  | |  | |  | | | **NS2 amino acid residues** | | | | | | | | | | | | | | |
| --- | --- | --- | --- | --- | --- | --- | --- | --- | --- | --- | --- | --- | --- | --- | --- | --- | --- | --- | --- | --- | --- | --- | --- | --- | --- |
| **Type/Variant^a^** | **Sample Id** | **Origin** | | **Country** | | **Year** | | **Acc.n.** | | | **60** | **81** | **93** | **94** | | **99** | | **105** | | **109** | | **110** | **151** | **152** | **160** |
| FPLV^b^ | CU-4 | Cat | | USA | | 1967 | | M38246 | | | I | V | D | T | | S | | R | | S | | D | D | M | E |
| FPLV | 42807/15 | Cat | | Italy | | 2015 | | KX434462 | | |  |  |  |  | |  | |  | |  | |  |  | V |  |
| FPLV | 3201c1/15 | Cat | | Italy | | 2015 | | KX434461 | | |  |  |  |  | |  | | S | |  | |  |  | V |  |
| FPLV | PA285c2/16 | Cat | | Italy | | 2016 | | MK413732 | | |  |  |  |  | |  | |  | |  | |  |  | V |  |
| FPLV | PA12880Fe/16 | Cat | | Italy | | 2016 | | MK413734 | | |  |  |  |  | |  | |  | |  | |  |  | V |  |
| FPLV | PA12880Mi/16 | Cat | | Italy | | 2016 | | MK413736 | | |  |  |  |  | |  | |  | |  | |  |  | V |  |
| FPLV | PA12880Re/16 | Cat | | Italy | | 2016 | | MK413735 | | |  |  |  |  | |  | |  | |  | |  |  | V |  |
| FPLV | 38056c2/15 | Cat | | Italy | | 2015 | | MK413727 | | |  |  |  |  | |  | |  | |  | |  |  | V |  |
| FPLV | 52333eva/15 | Cat | | Italy | | 2015 | | MK413729 | | |  |  |  |  | |  | |  | |  | |  |  | V |  |
| FPLV | 55611/15 | Cat | | Italy | | 2015 | | MK413730 | | |  |  |  |  | |  | | S | |  | |  |  | V |  |
| FPLV | 58774/15 | Cat | | Italy | | 2015 | | MK413731 | | |  |  |  |  | |  | |  | |  | |  |  | V |  |
| FPLV | PA11334/17 | Cat | | Italy | | 2017 | | MK413737 | | |  |  |  |  | |  | |  | |  | |  |  | V |  |
| FPLV | RG21/16 | Cat | | Italy | | 2016 | | MK413733 | | |  |  |  |  | |  | | S | |  | |  |  | V |  |
| FPLV | CT1375/17 | Cat | | Italy | | 2017 | | MK413738 | | |  |  |  |  | |  | |  | |  | |  |  | V |  |
| FPLV | 32369/15 | Cat | | Italy | | 2015 | | MK413728 | | |  |  |  |  | |  | |  | |  | |  |  | V |  |
| FPLV | 72752/13 | Cat | | Italy | | 2013 | | MK413724 | | |  |  |  |  | |  | |  | |  | |  |  | V |  |
| FPLV | 149/15 | Cat | | Italy | | 2015 | | MK413726 | | |  | I |  |  | |  | | S | |  | |  |  | V |  |
| FPLV | 4311/14 | Cat | | Italy | | 2014 | | MK413725 | | |  |  |  |  | |  | |  | |  | |  |  | V |  |
|  |  |  | |  | |  | |  | | |  |  |  |  | |  | |  | |  | |  |  |  |  |
| CPV-2^b^ | CPV-N | Dog | | USA | | 1978 | | M19296 | | | I | V | D | T | | S | | R | | S | | D | D | V | E |
| new CPV-2a | 29451/09 | Dog | | Italy | | 2009 | | KX434454 | | |  |  |  |  | |  | |  | |  | |  |  |  | Q |
| new CPV-2a | 987/10 | Dog | | Italy | | 2010 | | KX434457 | | |  |  | G |  | |  | |  | |  | |  |  |  |  |
| new CPV-2a | PA40697/16 | Dog | | Italy | | 2016 | | MK413739 | | |  |  |  |  | |  | |  | |  | |  | N |  |  |
| new CPV-2a | PA43847/16 | Dog | | Italy | | 2016 | | MG434738 | | |  |  |  |  | |  | |  | |  | |  | N |  |  |
| new CPV-2a | PA48686/16 | Dog | | Italy | | 2016 | | MG434739 | | |  |  |  |  | |  | |  | |  | |  | N |  |  |
| new CPV-2a | PA3213/17 | Dog | | Italy | | 2017 | | MG434740 | | |  |  |  |  | |  | |  | |  | |  | N |  |  |
| new CPV-2a | PA5610/17 | Dog | | Italy | | 2017 | | MG434741 | | |  |  |  |  | |  | |  | |  | |  | N |  |  |
| new CPV-2a | PA10388/17 | Dog | | Italy | | 2017 | | MG434742 | | |  |  |  |  | |  | |  | |  | |  | N |  |  |
| new CPV-2a | PA13577/17 | Dog | | Italy | | 2017 | | MG434743 | | |  |  |  |  | |  | |  | |  | |  | N |  |  |
| new CPV-2a | PA13579id90/17 | Dog | | Italy | | 2017 | | MG434744 | | |  |  |  |  | |  | |  | |  | |  | N |  |  |
| new CPV-2a | PA13579id93/17 | Dog | | Italy | | 2017 | | MG434745 | | |  |  |  |  | |  | |  | |  | |  | N |  |  |
| new CPV-2a | PA30636/17 | Dog | | Italy | | 2017 | | MK413740 | | |  |  |  |  | |  | |  | | F | |  | N |  |  |
| new CPV-2a | PA31209/17 | Dog | | Italy | | 2017 | | MK413741 | | |  |  |  |  | |  | |  | |  | |  | N |  |  |
| CPV-2b | PA13600/17 | Dog | | Italy | | 2017 | | MK413742 | | |  |  |  |  | | F | |  | |  | |  | N |  |  |
| CPV-2c | 23782/09 | Dog | | Italy | | 2009 | | KX434455 | | |  |  |  | A | |  | |  | |  | |  | N |  |  |
| CPV-2c | 45361/09 | Dog | | Italy | | 2009 | | KX434456 | | |  |  |  | A | |  | |  | |  | |  | N |  |  |
| CPV-2c | 25835/09 | Dog | | Italy | | 2009 | | KU508407 | | |  |  |  | A | |  | |  | |  | |  | N |  |  |
| CPV-2c | 2323/11 | Dog | | Italy | | 2011 | | KX434458 | | |  |  |  | A | |  | |  | |  | |  | N |  |  |
| CPV-2c | 27692c1/11 | Dog | | Italy | | 2011 | | KX434459 | | |  |  |  | A | |  | |  | |  | |  | N |  |  |
| CPV-2c | 52238/12 | Dog | | Italy | | 2012 | | KX434460 | | |  |  |  | A | |  | |  | |  | |  | N |  |  |
| CPV-2c | PA15423/16 | Cat | | Italy | | 2016 | | MK413743 | | |  |  |  | A | |  | |  | |  | | N | N |  |  |
| CPV-2c | PA39667/16 | Dog | | Italy | | 2016 | | MK413745 | | |  |  |  | A | |  | |  | |  | |  | N |  |  |
| CPV-2c | 41113c1/16 | Dog | | Italy | | 2016 | | MF510158 | | |  |  |  | A | |  | |  | |  | | N | N |  |  |
| CPV-2c | PA45984/16 | Dog | | Italy | | 2016 | | MK413747 | | |  |  |  | A | |  | |  | |  | |  | N |  |  |
| CPV-2c | PA36395/16 | Dog | | Italy | | 2016 | | MK413744 | | |  |  |  | A | |  | |  | |  | |  | N |  |  |
| CPV-2c | PA41113c2/16 | Dog | | Italy | | 2016 | | MK413746 | | |  |  |  | A | |  | |  | |  | |  | N |  |  |
| CPV-2c | 2743/17 | Dog | | Italy | | 2017 | | MF510157 | | | V |  |  |  | |  | |  | |  | |  | N |  |  |
| CPV-2c | CT1839id0018/17 | Dog | | Italy | | 2017 | | MK413748 | | |  |  |  | A | |  | |  | |  | |  | N |  |  |
| CPV-2c | CT1839id2213/17 | Dog | | Italy | | 2017 | | MK413749 | | |  |  |  | A | |  | |  | |  | |  | N |  |  |
| CPV-2c | PA27184/17 | Dog | | Italy | | 2017 | | MK413750 | | |  |  |  | A | |  | |  | |  | |  | N |  |  |

**^a^**The term “new” was used to distinguish the CPV-2a/2b strains with S297A VP2 amino acid change from the early CPV-2a/2b variants. ^b^reference strains.
