# Supplementary material for: Molecular Characterization and Evolutionary Analyses of Carnivore Protoparvovirus 1 NS1 Gene
Source: Viruses. 2019 Mar 29;11(4):308. doi: 10.3390/v11040308 (PMC6520740; doi:10.3390/v11040308)
Supplement: Supplementary file 1 [file viruses-11-00308-s001.zip › Recombination S5.docx]

**Supplementary Materials: Recombination S5.**  Phylogenetic reconstructions of the complete NS1 gene of CPV-2 and FPLV strains based on different genomic regions located upstream and downstream the putative recombination breakpoint (nt 1515). Evolutionary histories were inferred with the maximum-likelihood method based on the Hasegawa-Kishino-Yano and Kimura 2-parameters models, the best fitting models after the model test analysis, using MEGA 7. A discrete Gamma distribution was used to model evolutionary rate differences among sites. The outcome of the bootstrap analysis is shown next to the nodes and branch lengths are proportional to genetic distances as indicated by the scale bars. The recombinant strain is highlighted in red, the major parent in yellow and the minor parent in blue.
