# Supplementary figures and images for: Molecular Characterization and Evolutionary Analyses of Carnivore Protoparvovirus 1 NS1 Gene
Source: Viruses. 2019 Mar 29;11(4):308. doi: 10.3390/v11040308 (PMC6520740; doi:10.3390/v11040308)

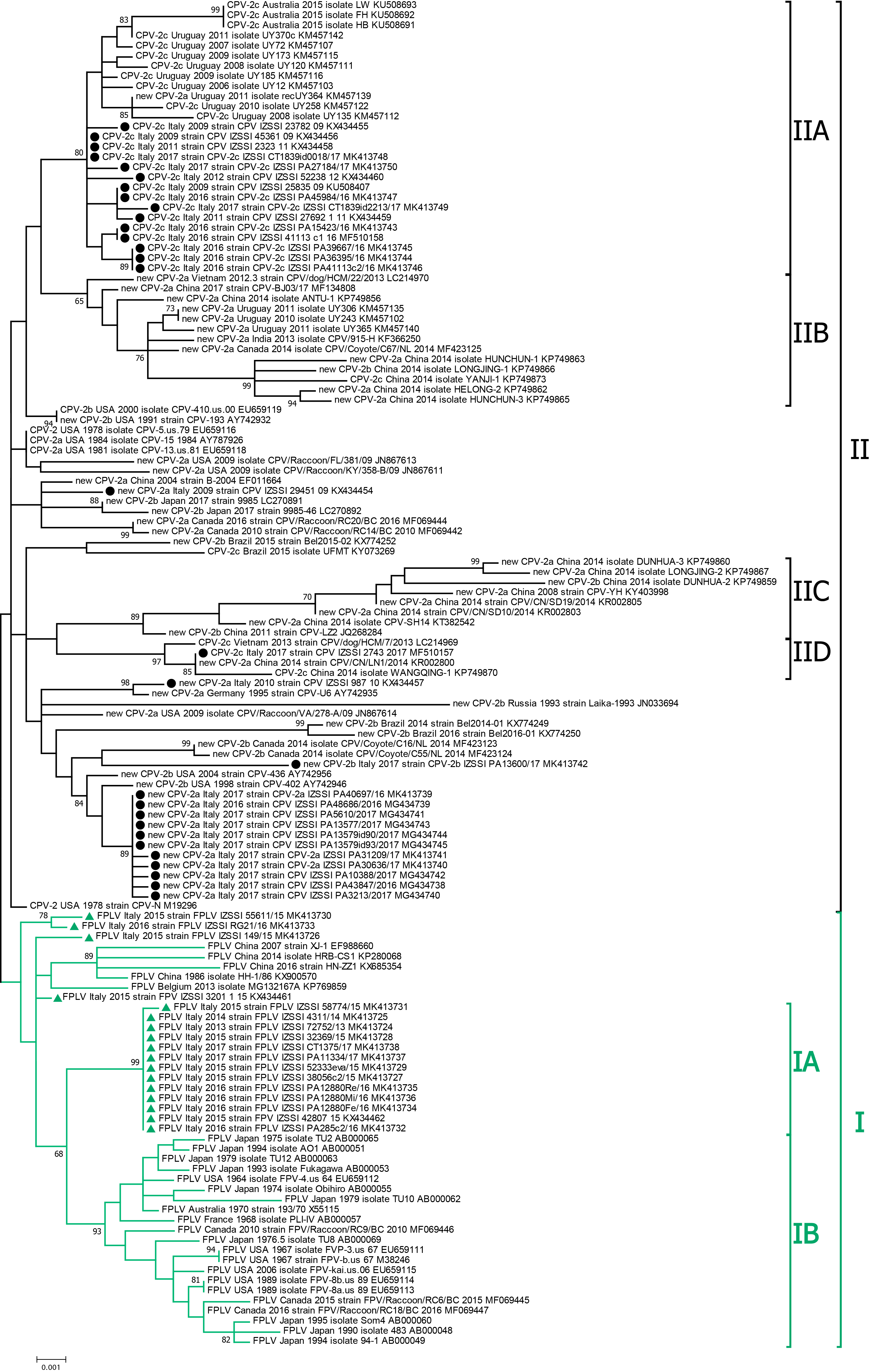

Supplement: Supplementary file 1 [file viruses-11-00308-s001.zip › Supplementary figure S6.jpg]

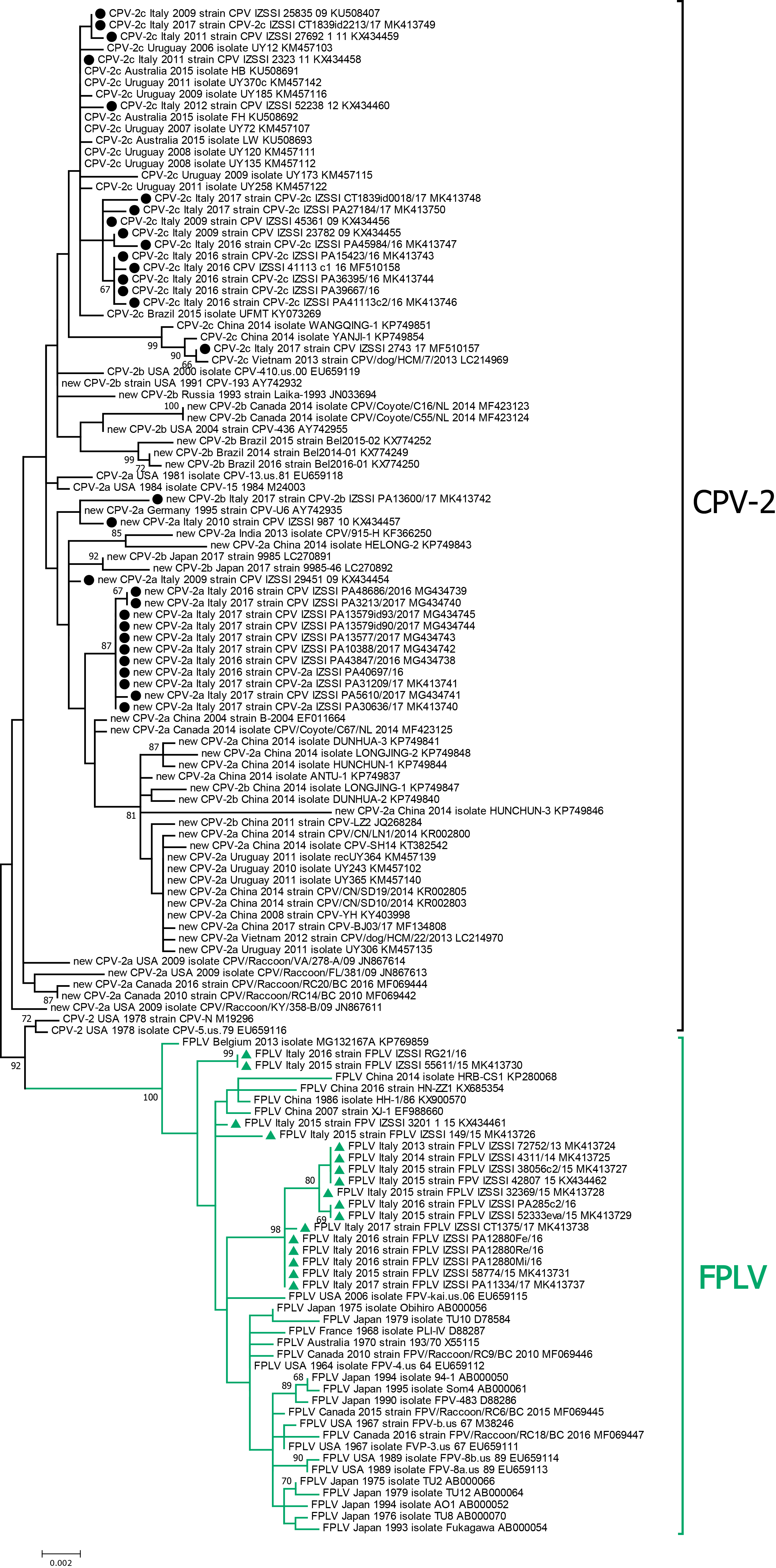

Supplement: Supplementary file 1 [file viruses-11-00308-s001.zip › Supplementary figure S7.jpg]
